# RRM2 silencing suppresses malignant phenotype and enhances radiosensitivity via activating cGAS/STING signaling pathway in lung adenocarcinoma

**DOI:** 10.1186/s13578-021-00586-5

**Published:** 2021-04-15

**Authors:** Xueping Jiang, Yangyi Li, Nannan Zhang, Yanping Gao, Linzhi Han, Shuying Li, Jiali Li, Xingyu Liu, Yan Gong, Conghua Xie

**Affiliations:** 1 Department of Radiation and Medical Oncology, Zhongnan Hospital of Wuhan University, Wuhan, 430071 Hubei China; 2 Department of Biological Repositories, Zhongnan Hospital of Wuhan University, Wuhan, 430071 Hubei China; 3 Tumor Precision Diagnosis and Treatment Technology and Translational Medicine, Hubei Engineering Research Center, Zhongnan Hospital of Wuhan University, Wuhan, 430071 Hubei China; 4 Hubei Key Laboratory of Tumor Biological Behaviors, Zhongnan Hospital of Wuhan University, Wuhan, 430071 Hubei China; 5 Hubei Cancer Clinical Study Center, Zhongnan Hospital of Wuhan University, Wuhan, 430071 Hubei China

**Keywords:** RRM2, Radiotherapy, CGAS/STING pathway, Immune responses, Lung adenocarcinoma

## Abstract

**Background:**

As one of the most common malignancy, lung adenocarcinoma (LUAD) is characterized by low 5-year survival rate. This research aimed to investigate the effects of ribonucleotide reductase regulatory subunit M2 (RRM2) on malignant biological behaviors and activation of cGAS/STING pathway. We also explored the synergistic sensitization mechanisms of RRM2 and radiotherapy.

**Methods:**

Bioinformatic tools were used to evaluate the clinical significance of RRM2 in LUAD patients. The roles of RRM2 in malignant phenotype and DNA damage in LUAD cells were investigated with cell proliferation, colony formation, immunofluorescence, modified Boyden chamber and comet assays. The mouse models were used to evaluate the biological significance of RRM2 in vivo. Cytotoxic T cell infiltration was evaluated via flow cytometric analysis and immunohistochemistry staining in C57BL/6 mice. We also explored the synergistic effects of RRM2 silencing and radiation on LUAD cells with apoptosis assay and immunoblotting in vitro.

**Results:**

Bioinformatic analysis revealed that RRM2 had diagnostic values for LUAD patients. Higher levels of RRM2 predicted worse prognosis. RRM2 silencing inhibited LUAD cell proliferation, invasion and migration. RRM2 knockdown induced S phase arrest and DNA damage. RRM2 silencing induced cyclic GMP-AMP synthase (cGAS)/stimulator of interferon genes (STING) pathway, and the downstream targets were regulated in a STING-dependent manner. Knockdown of RRM2 suppressed tumor growth in the xenograft tumor models. RRM2 deficiency increased CD8 + T cells in the tumor tissues and spleens. Furthermore, RRM2 silencing had synergistic effects with radiation on inhibiting cell proliferation and promoting apoptosis. Meanwhile, this combination promoted the activation of cGAS/STING signaling pathway synergistically, and simultaneously increased expression of IFNβ, CCL5 and CXCL10.

**Conclusion:**

Our results demonstrated that RRM2 silencing had anti-tumor values and activated the cGAS/STING signaling pathway. RRM2 silencing increased CD8 + T cells infiltration. RRM2 silencing cooperated with radiation to inhibit LUAD cell proliferation, promote apoptosis and enhance the activation of cGAS/STING signaling pathway. RRM2 could be a promising target for tumor regression through cancer immunotherapy in LUAD.

**Supplementary Information:**

The online version contains supplementary material available at 10.1186/s13578-021-00586-5.

## Introduction

Lung cancer is one of the most common cancers, and remains the first in terms of high mortality worldwide [1]. According to histopathologic characteristics, lung cancers are classified into small cell and non-small cell lung cancer (NSCLC). NSCLC is divided into 3 major types: adenocarcinoma (~ 50%), squamous cell carcinoma (~ 40%), and large cell carcinoma (~ 10%) [2]. Lung adenocarcinoma (LUAD) is characterized by low 5-year survival rate [3, 4]. Thus, it is critical to explore novel biomarkers and formulate integrated treatment strategies to improve prognosis of the patients.

Radiotherapy triggers immune responses and has significant anti-tumor effects during LUAD treatment [5]. However, the molecular mechanisms remain poorly understood. Previous researches proved that cytoplasmic DNA sensing mediated cyclic GMP-AMP synthase (cGAS)/stimulator of interferon genes (STING) signaling pathway could be one of the reasons to explain this specific immune effects. Ionizing radiation induces double-strand breaks (DSBs), and then the DNA fragments, which leak through the damaged nuclear envelope, enhance the accumulation of double-stranded DNA (dsDNA) in cytoplasm [6]. These dsDNA sensed by cGAS trigger the transduction of cGAS/STING signaling pathway, which promotes the activation of CD8 + cytotoxic T cell-mediated destruction of cancer [7, 8]. The accumulation of cytoplasmic dsDNA induced by DNA damage initiates immune responses. Activation of cGAS/STING pathway is a potential strategy to improve therapeutic effects of immunotherapy.

Ribonucleotide reductase (RNR) is important for accurate DNA replication and repair via producing deoxyribonucleotide triphosphate (dNTP), and further affects the stability of genome [9]. The de novo dNTP biosynthesis is dependent on the RNR activity in cancer cells. Human RNR is composed of 2 kinds of subunits, α subunit encoded by *RRM1*, and β subunit encoded by *RRM2* or *p53R2* [9]. The tumorigenesis and development of cancers are closely linked to uncontrolled cell proliferation [10]. During the whole cell cycle, RRM1 and p53R2 remain continuously expressed. RRM2 controls the cell cycle-dependent activity of RNR, and its expression is regulated by both transcription and protein degradation [11, 12].

Previous studies proved that RRM2 played an oncogenic role in multiple cancers. Overexpressed RRM2 promotes invasiveness in gastric cancer [13] and inhibits cell apoptosis in human glioblastoma [14]. RRM2 was also reported to be related to endogenous DNA damage and repair. In osteosarcoma cells, RRM2 silencing significantly reduced the homologous recombination (HR) activity [15]. In primary effusion lymphoma cells, RRM2 knockdown induced DNA damage and promoted the phosphorylation of γH2AX at the Ser 139 site [16], which was the indicator of DSBs [17, 18]. However, very little is known about the effects of RRM2 on malignant biological behaviors and downstream signaling transduction in LUAD.

In the present study, we addressed that RRM2 silencing inhibited cell proliferation, migration, invasion, and induced S phase arrest. Moreover, targeting RRM2 induced DNA damage and activated cGAS/STING signaling pathway. Knockdown of RRM2 increased CD8 + T cells infiltration in vivo. We also detected that RRM2 silencing enhanced radiosensitivity of LUAD via synergistically enhancing the transduction of cGAS/STING signaling pathway.

## Materials and methods

### Bioinformatic analysis

The LUAD patients’ transcription profiles and clinical data were obtained from TCGA GDC website [19]. The Kaplan–Meier (K-M) survival curve analysis was used to compare different overall survival between the high- and low-expression groups in TCGA dataset. The Gene Set Enrichment Analysis (GSEA) was conducted with basic document named c2.cp.kegg.v7.0.symbols.gmt, which was downloaded from molecular signatures database (https://www.gsea-msigdb.org/gsea/datasets.jsp). The number of permutations was set as 1,000 times. The enriched results were considered as significant that satisfied both of the following criteria simultaneously: 1) Nominal *p* < 0.05, 2) FDR *q* < 0.05. The concordance index was calculated using the coxph function of “survival” R package. The univariate logistic regression analysis was used to evaluate the relevance between RRM2 expression and clinical features. The multivariate Cox regression analysis was applied to further validate the expression of RRM2 as an independently prognostic factor. The online tool TIMER[20] was utilized to explore the correlation between RRM2 and STING expression in LUAD.

### Cell culture and irradiate treatment

A549, PC9, H1299, H1975 and Beas-2B cells were cultured in RMPI 1640 medium with 10% fetal bovine serum (FBS) in humidified incubator (37 °C, 5% CO2). The Lewis lung carcinoma (LLC) cells were cultured in DMED medium with 10% FBS in humidified incubator (37 °C, 5% CO2). The fluorescent staining of mycoplasma in LUAD cells was presented in Additional file 1: Fig. S1.

### RNA interference and lentiviral transfection

Small interfering RNAs (siRNAs) and negative control (NC) were transfected at 20 nM using jetPRIME® transfection reagent. A549 and LLC cells were infected with NC or siRNA lentiviruses (LV) at optimal multiplicity of infection. The stably RRM2-deficient cells were selected with puromycin (4.5 μg/mL for A549; 4 μg/mL for LLC). The targeting siRNA sequences were presented in Additional file 1: Table S1.

### RNA isolation and quantitative real-time PCR

Total RNA was isolated from cells using TRIZOL. The RNA concentrations and quality were evaluated by a Nanodrop spectrophotometer. Total RNA (1 μg) was reversely transcripted using TRUEscript 1^st^ Strand cDNA Synthesis Kit With gDNA Eraser. Quantitative real-time PCR (qRT-PCR) was performed using the SG qPCR Mix in the CFX Connect™ RT-PCR Detection System. The relative expression fold changes of mRNAs were calculated by the 2 − ΔΔCt method. Primer sequences were presented in Additional file 1: Table S1.

### Protein isolation and immunoblotting

Protein was extracted from the cells using RIPA cell lysis buffer supplemented with phosphatase and protease inhibitors. The cells were lysed on ice for 30 min and then centrifugated (12,000 rpm, 4 °C) for 15 min. The proteins were electrophoresed in SDS-PAGE gel and transferred to PVDF membrane. After blocking in 5% skimmed milk for 2 h at room temperature (RT), the membranes were incubated with primary antibodies at 4 °C overnight. After washing, the membranes were incubated with secondary antibodies at RT for 1 h. Bands were detected with high sensitivity electrochemiluminescence detection kit and captured with chemiluminescence imaging system. Relative protein expression was quantified with ImageJ software. The antibodies were presented in Additional file 1: Table S2.

### Cell proliferation and colony forming assay

For CCK8 assay, cells (3 × 10^3^ cells/well) were seeded into 96-well plates and cultured for 24 h. After discarding culture medium, 100 ul CCK8 solution was added into each well. The optical density (OD, 450 nm) values were measured by SpectraMax® Absorbance Reader.

For colony forming assay, the cells were seeded into 6-well plates (1 × 10^3^ cells/well) 48 h after transfection. After culture for another 15 days, the colonies were fixed with paraformaldehyde (4%, RT, 30 min) and stained with crystal violet (0.5%, RT, 30 min). The number of colonies was counted under the light microscope.

### Modified Boyden chamber assay

The transfected cells (3 × 10^4^ cells/200 μl PRIM 1640) were added into the upper chamber. Culture medium (RMPI 1640 plus 10% FBS, 600 μl) were added into the lower chamber to induce cell invasion and migration. After culturing for another 24 h, cells on the upper surface of the polycarbonate membrane were completely wiped. The cells on the bottom of the upper chamber were fixed with paraformaldehyde (4%, RT, 30 min) and stained with crystal violet (0.5%, RT, 30 min). Three random fields were selected under the light microscope, and the number of the invading and migrating cells were counted using the ImageJ software.

### Flow cytometry

For cell cycle, the harvested cells were incubated with DNA staining and permeabilization solution (away from light, RT, 30 min). The samples were tested on CytoFLEX system and analyzed by Modifit software.

For apoptosis analysis, the harvested cells were resuspended gently with binding buffer and Annexin V-FITC staining solution (away from light, 4 °C, 15 min). After incubating with propidium iodide (PI) solution for another 5 min, the cells were analyzed on CytoFLEX system.

For the investigation of cytotoxic T cell infiltration, single-cell suspensions were prepared and the cells were stained with fluorescence-labeled antibodies against CD3, CD45, CD4 and CD8 (BD Pharmingen). The data were acquired on FACS Aria TM III Cell Sorter and analyzed with FlowJo (version 10.7.1). The antibodies were presented in Additional file 1: Table S2.

### Immunofluorescence, immunohistochemistry, and hematoxylin and eosin staining

For immunofluorescence (IF), the cells on 24 × 24 mm glass slides were fixed with paraformaldehyde (4%, RT, 30 min), and then permeabilized with Triton X − 100 (0.5%, RT, 20 min). After washing, the cells were blocked with bovine serum albumin (5%, RT, 1 h), and then incubated with primary antibodies (Additional file 1: Table S2, 4 °C, 24 h). After washing with Tween20 (0.1%, 3 times, 15 min), cells were incubated with secondary antibodies (away from light, RT, 1 h). The images were taken by fluorescent or confocal microscope. For the xenograft tumor tissues, IF, immunohistochemistry (IHC) and hematoxylin and eosin (H&E) staining was performed by Biofavor Biotech, China.

### Comet assay

The comet assay was performed using single cell gel electrophoresis kit. Briefly, the transfected cells were immobilized on the comet slide using low melting agarose, lysed at 4 °C for 2 h, followed by electrophoresis at 25 V for 30 min in alkaline electrophoretic buffer (1 mmol/L EDTA, 300 mmol/L NaOH). Gels were then neutralized with Tris–HCl buffer (0.4 mmol/L, PH = 7.5, 3 times, 10 min) and stained with PI. Cells were photographed using a fluorescent microscope, and the comet tails were analyzed by CASP software.

### Enzyme-linked immunosorbent assay

The culture medium was collected from LUAD cells 48 h after transfection. The levels of IFNβ, CCL5 and CXCL10 cytokines were analyzed with enzyme-linked immunosorbent assay (ELISA) kits. The OD 450 nm values were measured by SpectraMax® Absorbance Reader.

### Xenograft tumor mouse model

The BALB/c nude mice (5 weeks, male) and C57BL/6 mice (5 weeks, male) were purchased from the Jiaxing Wanqian Biology Technology. For nude xenograft tumor model, the stable A549-LV-NC or A549-LV-siRRM2 cells (4 × 10^7^ cells/100 μl for each mouse) were subcutaneously injected into the right armpits. After 6 weeks, mice were sacrificed and tumors were isolated for further research.

For C57BL/6 xenograft tumor model, the stable LLC-LV-NC or LLC-LV-siRRM2 cells (1 × 10^7^ cells/100 μl for each mouse) were subcutaneously injected into the right armpits. After 4 weeks, mice were sacrificed and tumors were isolated for further research. The volume of the tumors was detected by IVIS Spectrum in vivo imaging system. Radiance values of the tumor were calculated automatically by the imaging system with a standard circle overlaying the tumor area.

The volume of tumor was also monitor by manual measurement and calculated according to the following formula every 3 days.

$$ V\left( {mm^{3} } \right) = \frac{length \times width \times width}{2} $$

The animal experiment was approved by the Institutional Animal Care and Use Committee at Center for Animal Experiment, Wuhan University.

### Statistical analysis

All the data were processed with R (version: 3.6.0) and GraphPad Prism (version: 5.0). All quantitative results were shown as the mean ± standard deviation. The unpaired student's *t*-test and one-way analysis of variance were used to comparison the difference between 2 groups or more. Correlations were analyzed by Spearman correlation test. K-M survival analysis was used log-rank test. The differential expression levels of RRM2 were analyzed by Wilcoxon rank sum test. The correlation of RRM2 expression with clinical characteristics was analyzed by Kruskal–Wallis rank sum test. A value of *p* < 0.05 was considered as statistical significance.

## Results

### The significantly high expression of RRM2 is associated with LUAD clinical features

**Fig. 1:**
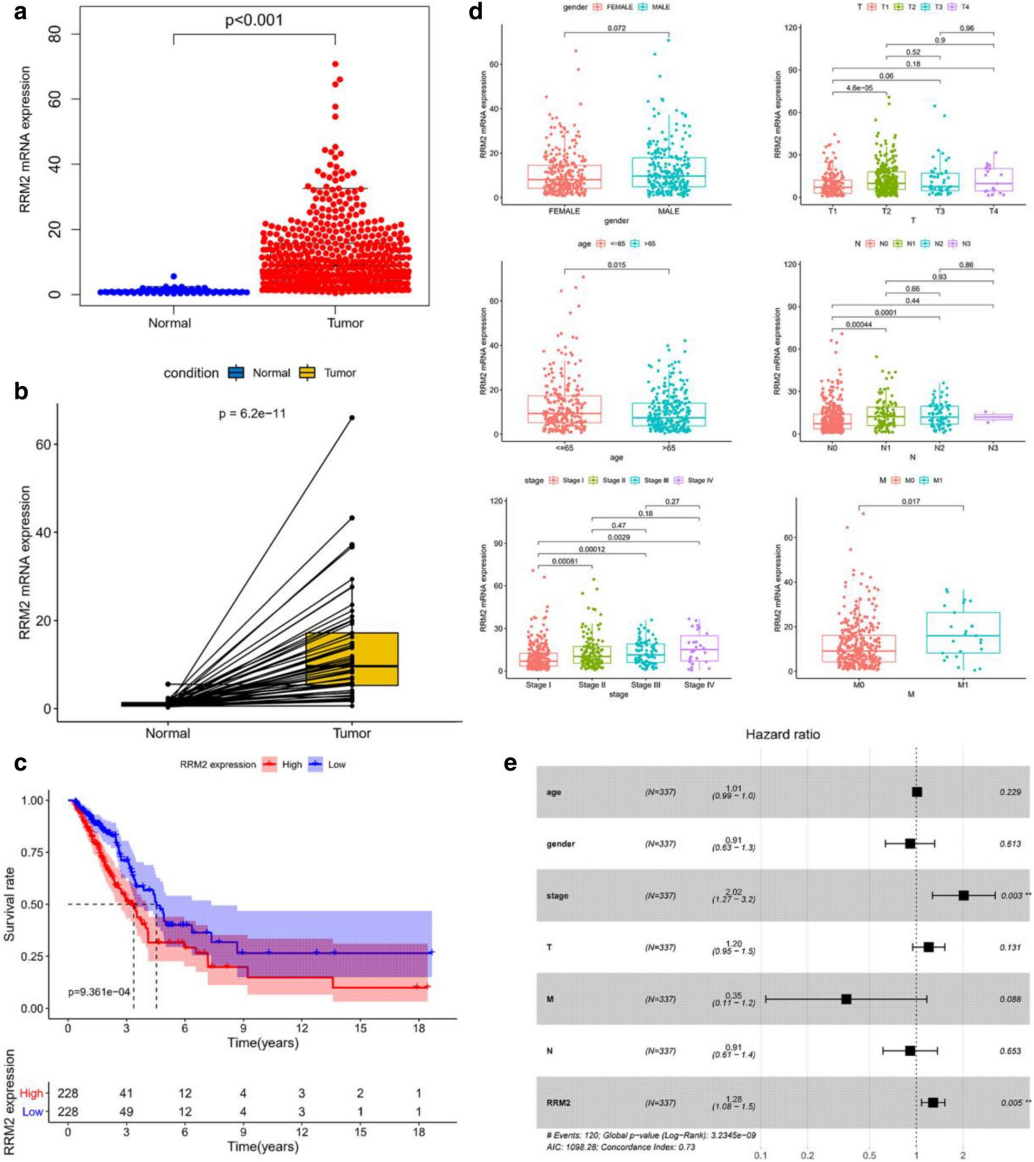
The high expression of RRM2 was correlated with worse prognosis and clinicopathological staging characteristics for LUAD patients. **a** The expression of RRM2 was much higher in tumor samples. **b** The expression of RRM2 was significantly increased in the tumor tissues compared with that in the adjacent non-cancerous tissues. **c** The K-M survival curve analysis for LUAD patients based on RRM2 expression. **d** The correlation between RRM2 expression and clinical features. **e** RRM2 was confirmed to be an independent prognostic element with multivariate cox regression model

To analyze the expression of RRM2 in LUAD patients, the transcriptome sequencing data (n = 594) were downloaded from TCGA database. The relative mRNA levels of RRM2 in tumor samples were much higher than those in normal samples, and also much higher in tumor tissues than those in corresponding adjacent nontumorous tissues (Fig. 1a, b). According to the median value, the TCGA-LUAD patients were divided into the RRM2 high- and low-expression groups. The patients in the low-expression group had longer survival than those in the high-expression group (Fig. 1c). Moreover, RRM2 expression was strongly associated with clinical features based on TCGA-LUAD database. The RRM2 expression was lower in younger patients (≤ 65 years). Higher RRM2 expression tended to be associated with worse clinicopathological features including high TNM stages. Furthermore, the RRM2 expression did not differ between genders (Fig. 1d). Univariate logistic regression indicated that high RRM2 expression was correlated with poor prognosis clinical characteristics (Table 1), suggesting that LUAD patients with high RRM2 levels tended to progress to a more advanced stage. Multivariate cox regression indicated that high RRM2 expression [Hazard ratio (HR) = 1.28, 95% Confidence interval (CI): 1.08–1.5, *p* = 0.005] and high stage (HR = 2.02, 95% CI: 1.27–3.2, *p* = 0.003) were significantly associated with the prognosis for LUAD patients (Fig. 1e). These results indicated that RRM2 played oncogenic roles and predicted the prognosis of LUAD patients.

**Table 1:** Association between RRM2 expression and clinicopathologic characteristics (logistic regression)

Clinical characteristics	Total (N)	Odds ratio (OR) in RRM2 expression	*p*-value
Age	494		
≤ 65 vs. > 65		0.7464644 (0.5234463–1.06287)	0.1053727
Gender	513		
Male vs. Female		1.357798 (0.9591132–1.925223)	0.08510176
Stage	505		
II vs. I		1.55754 (1.014021–2.399717)	0.04346904*
III vs. I		2.180556 (1.327597–3.627003)	0.002300152**
IV vs. I		3.019231 (1.30853–7.578007)	0.01244337*
T	510		
T2 vs. T1		1.959701 (1.329787–2.902402)	0.0007191007***
T3 vs. T1		1.326567 (0.6885868–2.545255)	0.3947867
T4 vs. T1		1.674959 (0.6424348–4.427256)	0.2882927
N	501		
N2 vs. N1		1.75431 (1.108522–2.796568)	0.0170222*
N3 vs. N1		2.50069 (1.487095–4.295968)	0.0006760279***
N4 vs. N1		1.275862 (0.05016816–32.44753)	0.8636424
M	369		
M1 vs. M0		2.252246 (0.9743585–5.647803)	0.06628612

### RRM2 silencing inhibits cell proliferation and induces S phase arrest

**Fig. 2:**
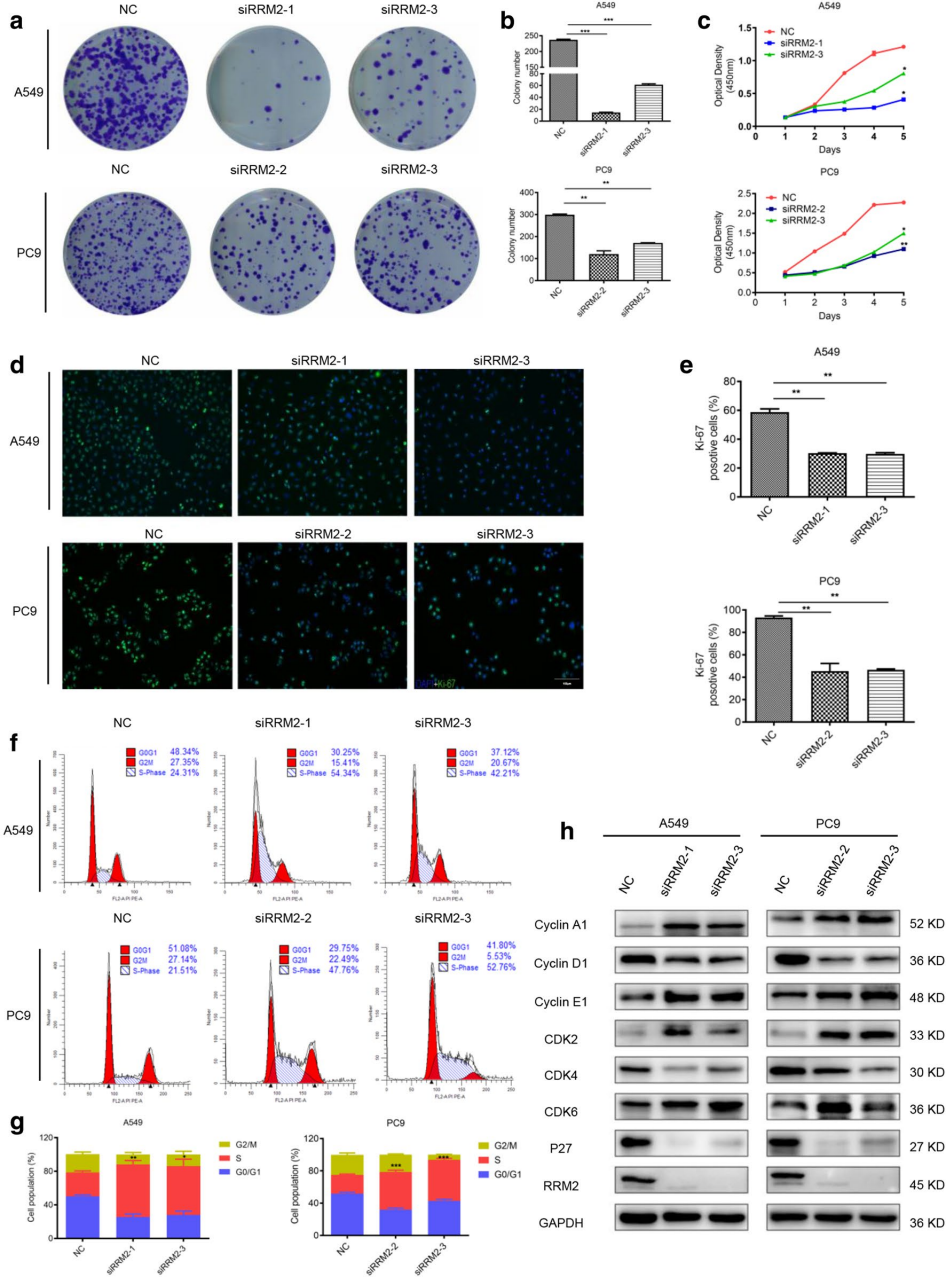
RRM2 silencing inhibited cell proliferation and induced S phase arrest. **a** Colony forming assay indicated the decreased numbers of colonies after transfecting siRRM2 in A549 and PC9 cells. **b** Statistical graphs of colony formation assay. **c** CCK8 assay showed that RRM2 silencing significantly inhibited A549 and PC9 cell proliferation. **d** Representative images of Ki-67 immunofluorescent staining of RRM2-deficient cells. Scale bar: 100 μm. **e** Quantification of Ki-67 positive cells. **f**, **g** A549 and PC9 cells were analyzed by flow cytometry for cell cycle 48 h after transfection. **h** Immunoblotting of the cell cycle related proteins, GAPDH was used as an internal control. All tests were repeated 3 times. *p < 0.05, **p < 0.01, ***p < 0.001

The differential expression of RRM2 in LUAD cell lines (A549, PC9, H1299 and H1975) were detected by immunoblotting (Additional file 1: Fig. S2). RRM2 expression levels were much higher in A549 and PC9 cells than normal Beas-2B cells. To knockdown RRM2, A549 and PC9 cells were transfected with siRNAs specifically targeting RRM2, and the efficiency of silencing was evaluated with qRT-PCR and immunoblotting (Additional file 1: Fig. S2). The results of colony forming assay indicated that RRM2 deficiency significantly decreased the colony numbers (Fig. 2a, b). Similarly, RRM2 knockdown significantly inhibited A549 and PC9 cell proliferation (Fig. 2c). Moreover, RRM2 silencing resulted in a significant reduction of proliferation marker Ki-67[21] positive cells (Fig. 2d, e). Flow cytometry was conducted to evaluate the effects of RRM2 on cell cycle distribution. Our results revealed that RRM2 silencing induced S phase arrest (Fig. 2f, g). The results of immunoblotting demonstrated upregulated protein levels of CyclinA1, CyclinE1, and CDK2/6, and downregulated protein levels of CyclinD1, CDK4 and P27 after RRM2 silencing (Fig. 2h). We concluded that RRM2 knockdown inhibited cell proliferation and induced S phase arrest in LUAD cells.

### RRM2 affects LUAD cell motility with the alteration of epithelial mesenchymal transition (EMT) related proteins

**Fig. 3:**
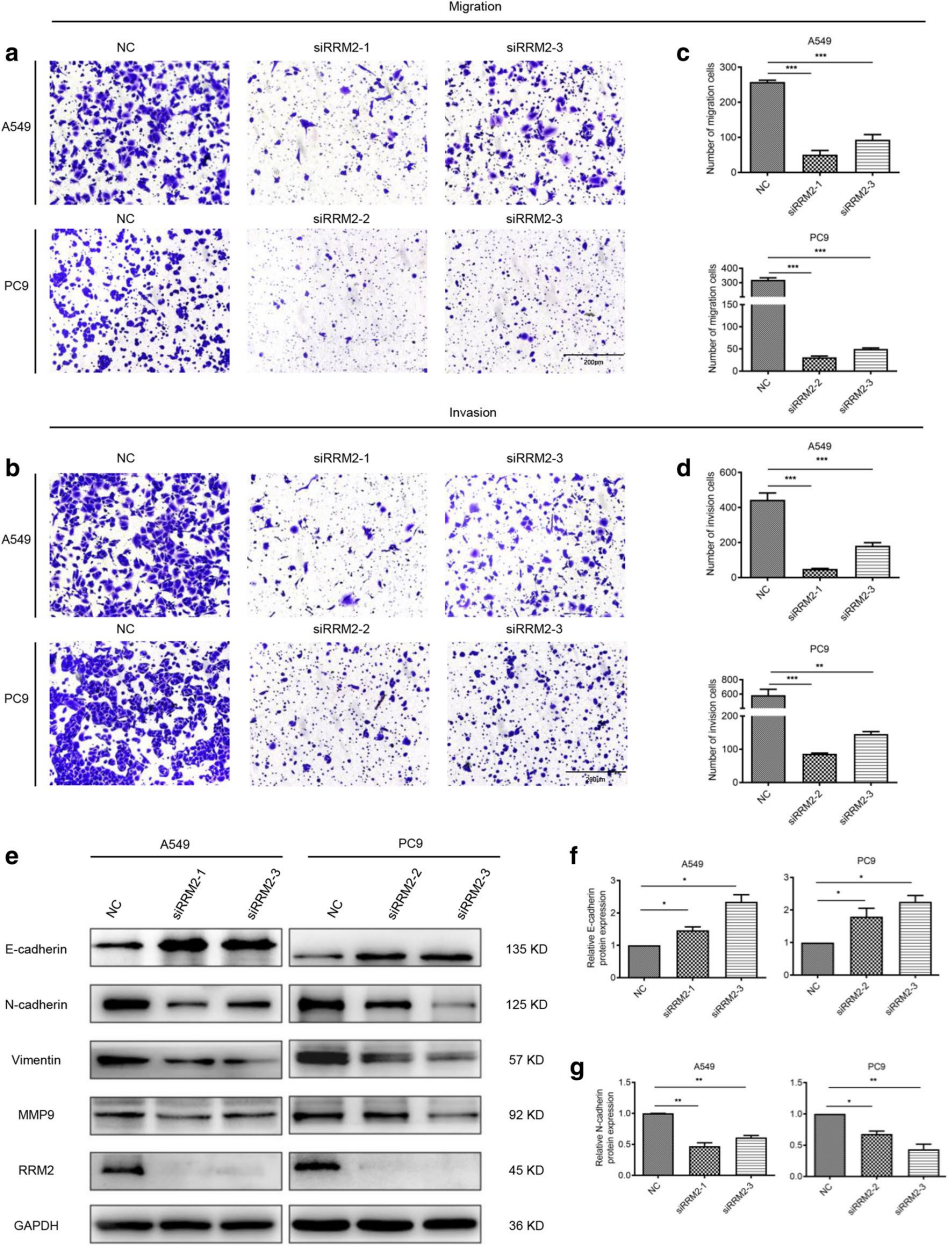
RRM2 silencing inhibited cell migration and invasion. **a** The effects of RRM2 silencing on cell migration were evaluated with transwell assay in A549 and PC9 cells. Scale bar: 200 μm. **b** Quantification of the cell number of migrated cells. **c** The effects of RRM2 silencing on cell invasion were evaluated by Matrigel transwell assay. Scale bar: 200 μm. **d** Quantification of the numbers of cells crossing the Matrigel (invasion). **e** Immunoblotting of the EMT-related proteins. GAPDH was used as an internal control. **f**, **g** Relative intensity of key EMT-related proteins including E-cadherin and N-cadherin in A549 and PC9 cells after knockdown of RRM2. All tests were repeated 3 times. *p < 0.05, **p < 0.01, ***p < 0.001

The effects of RRM2 silencing on cell migration and invasion was evaluated with modified Boyden chamber assay. After RRM2 knockdown, the capacity of migration and invasion were inhibited (Fig. 3a, b). Statistical analysis of 3 independent experiments confirmed the significant reduction of invasion and migration rates (Fig. 3c, d). The results of immunoblotting demonstrated that the protein levels of E-cadherin were increased, while the other biomarkers including N-cadherin, Vimentin and MMP9 were downregulated after RRM2 silencing (Fig. 3e–g). The results demonstrated that RRM2 deficiency inhibited the capacity of migration and invasion.

### RRM2 silencing induces DNA damage in LUAD cells

**Fig. 4:**
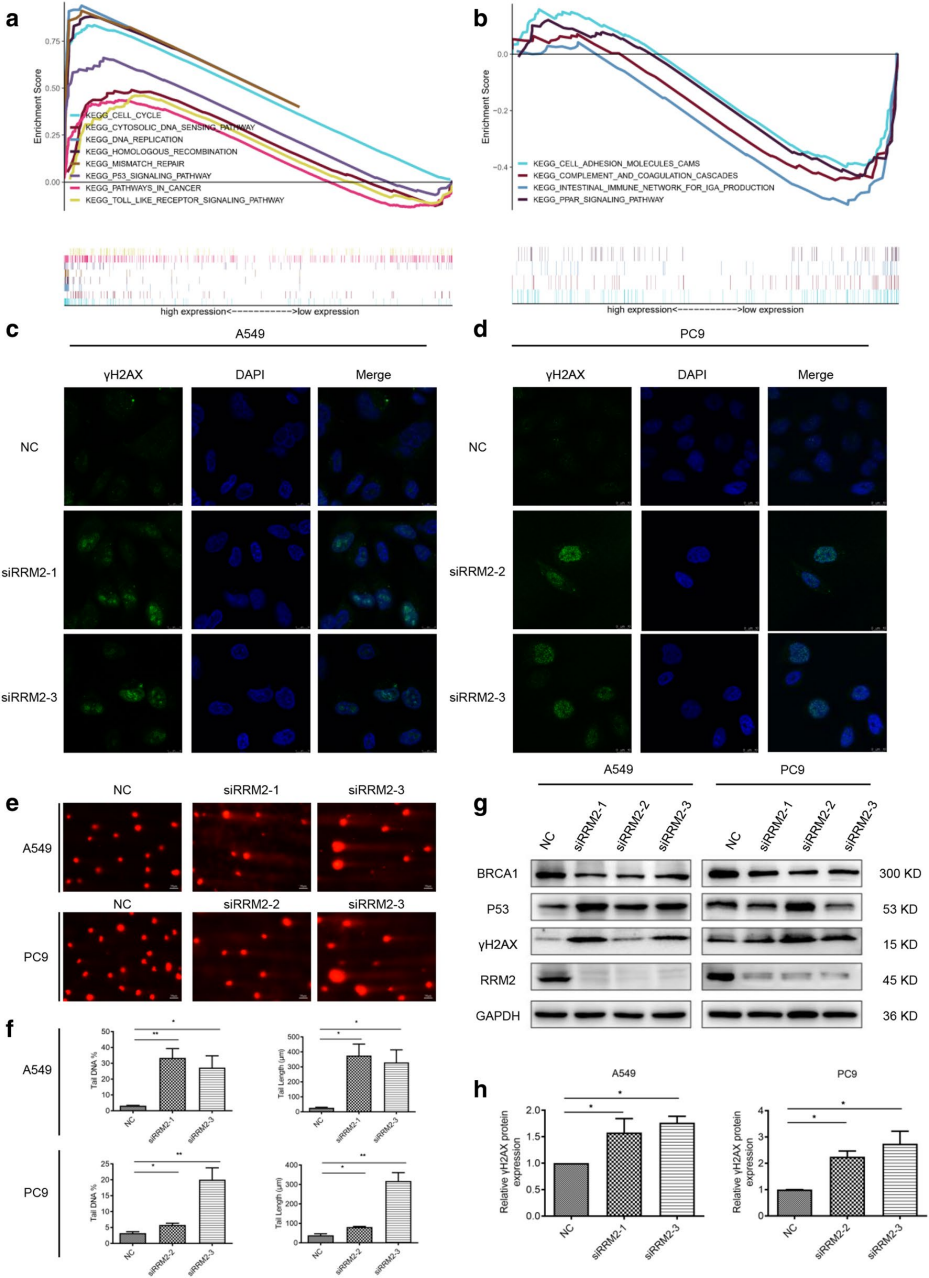
RRM2 silencing induced DNA damage in LUAD cells. **a**, **b** GSEA for the enriched gene sets in the RRM2 high- and low-expression groups. **c**, **d** immunofluorescence was performed to detect γH2AX foci formation. The representative images were taken with confocal microscope. Scale bar: 10 μm. **e**, **f** Comet assay was performed after transfecting siRRM2 into A549 and PC9 cells for 48 h. Representative pictures were shown. Scale bar: 50 μm. The percentage of tail DNA content and tail length of the comet was quantified and graphed. **g** Immunoblotting of BRCA1, P53 and γH2AX. GAPDH was used as an internal control. **h** Relative intensity of γH2AX in A549 and PC9 cells after RRM2 knockdown. All tests were repeated 3 times. *p < 0.05, **p < 0.01

To further investigate the underlying mechanisms, GSEA analysis was applied to explore the RRM2-related signaling pathways. The genes in the RRM2 high-expression group were mainly enriched in DNA-related signaling pathways including cell cycle, cytosolic DNA sensing pathway, DNA replication, HR, and p53 signaling pathways (Fig. 4a). In the RRM2 low-expression group, the enrichment signaling pathways were associated with cell adhesion molecules cams, complement and coagulation complement, and PARP signaling pathway (Fig. 4b). The details of GSEA results were shown in Additional file 1: Table S3.

Next, we examined the effects of RRM2 silencing on DNA damage in LUAD cells. Confocal microscopy (Fig. 4c, d) indicated that RRM2 silencing induced DNA damage with significant activation of DNA damage marker γH2AX. Moreover, the comet assay showed that A549 and PC9 cells treated with RRM2 siRNAs had significantly higher DNA damage than the control cells (Fig. 4e). The percentage of tail DNA content and tail length of the comets were increased significantly after RRM2 silencing in LUAD cells (Fig. 4f).

Meanwhile, we examined the protein levels of key genes related to DNA damage and repair by immunoblotting. The results suggested that RRM2 deficiency reduced BRCA1, and induced p53 and γH2AX (Fig. 4g, h). All the results demonstrated that RRM2 silencing aggravated DNA damage in LUAD cells.

### RRM2 silencing activates cGAS/STING signaling pathway

**Fig. 5:**
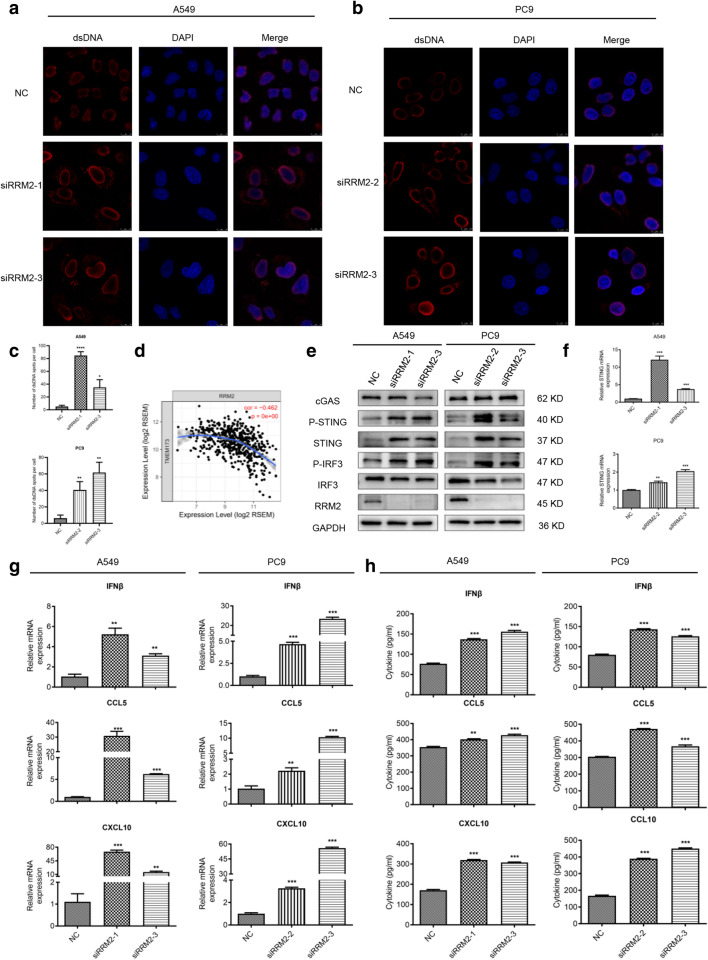
RRM2 silencing activated cGAS/STING signaling pathway. **a**, **b** Immunostaining was performed to determine the accumulation of dsDNA in cytoplasm. The representative images were taken with confocal microscope. Scale bar: 10 μm. **c** The numbers of dsDNA spots were quantified and graphed. **d** The correlation analysis between the expression of RRM2 and STING in LUAD patients was performed with TIMER database. **e** Immunoblotting of the classical cGAS/STING pathway proteins including cGAS, p-STING, STING, p-IRF3 and IRF3. GAPDH was used as an internal control. **f** The mRNA levels of STING were detected with qRT-PCR in A549 and PC9 cells after RRM2 silencing. **g** The mRNA levels of cGAS/STING downstream molecules (IFNβ, CCL5 and CXCL10) were detected by qRT-PCR in RRM2-deficient cells. **h** The secretion of IFNβ, CCL5 and CXCL10 were assayed by ELISA in RRM2-deficient cells. All tests were repeated 3 times. **p < 0.01, ***p < 0.001

GSEA results showed that RRM2 was related to cytosolic DNA sensing pathway such as cGAS/STING signaling pathway, we thus investigated the cytosolic dsDNA with confocal microscopy. The results demonstrated that RRM2 silencing increased the accumulation of dsDNA in cytosol (Fig. 5a, b). The statistical analyses were shown in Fig. 5c. Previous studies suggested that cGAS bound to dsDNA and then activated cGAS/STING pathway [22–24]. Moreover, our results demonstrated that the cytosolic dsDNA colocalized with cGAS, and that RRM2 deficiency increased the merged signals in LUAD cells (Additional file 1: Fig. S3). In TIMER database, the results suggested that RRM2 expression was negatively correlated with STING in LUAD samples (Fig. 5d). To validate this, immunoblotting was performed to detect the protein levels of STING. Our results indicated that RRM2 deficiency resulted in the increase of STING, and upregulation of phosphorylation of STING and interferon regulatory factor (IRF) 3, but there were no significant changes on cGAS levels (Fig. 5e, f). Moreover, qRT-PCR and ELISA assays revealed that RRM2 silencing markedly enhanced the production of IFNβ, CCL5 and CXCL10, which were the key downstream molecules of cGAS/STING pathway (Fig. 5g, h). RRM2 deficiency increased mRNA levels of IL-6, MX1 and ISG56 in A549 and PC9 cells (Additional file 1: Fig. S4). We concluded that knockdown RRM2 activated cGAS/STING signaling pathway in LUAD cells.

### The regulation of cGAS/STING signaling pathway by RRM2 is mediated by STING

**Fig. 6:**
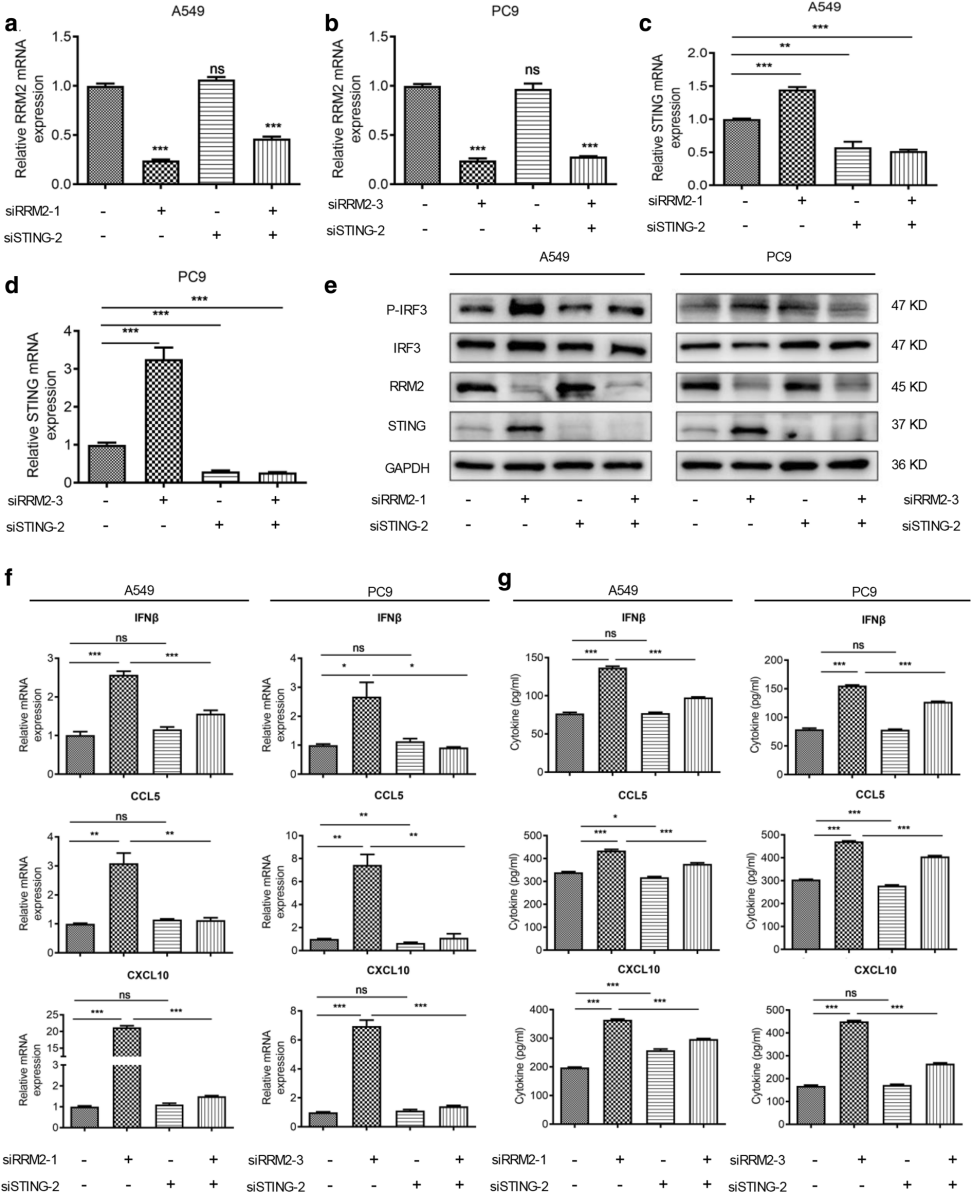
STING downregulation partially rescued the activation of cGAS/STING signaling pathway induced by RRM2 silencing. The mRNA levels of RRM2 in A549 (**a**) and PC9 (**b**) cells after transfecting siRRM2, siSTING or their combination. The mRNA levels of STING in A549 (**c**) and PC9 (**d**) cells after transfecting siRRM2, siSTING or their combination. (**e**) The effects of siRRM2, siSTING or their combination on cGAS/STING pathway were evaluated by immunoblotting. The mRNA levels (**f**) and the secretion (**g**) of IFNβ, CCL5 and CXCL10 in A549 and PC9 cells after transfecting siRRM2, siSTING or their combination. All tests were repeated 3 times. **p < 0.01, ***p < 0.001

To investigate whether RRM2 knockdown mediated the activation of cGAS/STING signaling pathway in a STING-dependent manner, STING was downregulated by siRNAs (Additional file 1: Fig. S5), and siSTING-2 was chosen for further studies due to its higher efficiency. While RRM2 was not regulated by STING in LUAD cells (Fig. 6a, b), the mRNA levels of STING were upregulated in RRM2 silencing cells, and could be restored in the siSTING groups (Fig. 6c, d). Furthermore, RRM2 silencing enhanced the phosphorylation of IRF3, and STING deficiency downregulated phospho-IRF3 (p-IRF3) (Fig. 6e). RRM2 silencing upregulated cGAS/STING downstream molecules, such as IFNβ, CCL5 and CXCL10, and these effects were partially inhibited by STING silencing (Fig. 6f, g). These data suggested that the regulation of cGAS/STING signaling pathway by RRM2 was mediated by STING.

### RRM2 silencing had anti-tumor effects in vivo

**Fig. 7:**
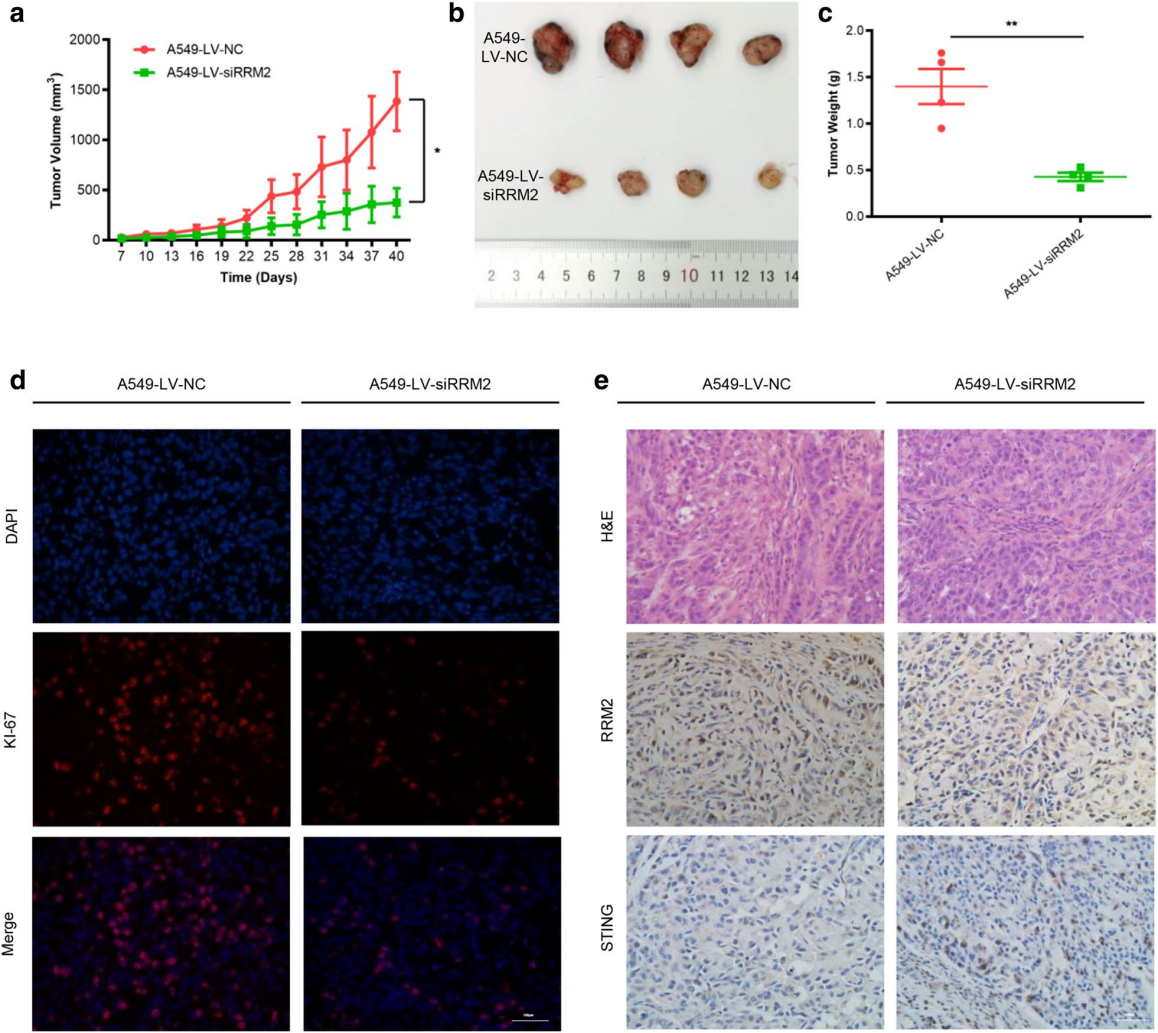
RRM2 silencing suppresses LUAD cells growth in nude mice model. Nude mice were injected with LV-NC and LV-siRRM2 infected A549 cells. **a** The tumor volumes were measured every 3 days and depicted in the line chart. **b** On the 40th day of injection, the tumors were collected for photograph. **c** The final weights of tumors were recorded. **d** Representative Ki-67 staining of tumor tissues. Scale bar: 100 μm. **e** Representative H&E staining and IHC (RRM2 and STING) of tumor tissues. Scale bar: 100 μm. *p < 0.05, **p < 0.01

To further investigate the effects of RRM2 silencing in vivo, nude mice were injected with LV-siRRM2 stably transfected A549 cells. RRM2 deficiency significantly suppressed tumor growth (Fig. 7a). The tumor weight of the LV-siRRM2 group was significantly lower than that of the LV-NC group (Fig. 7b, c). The percentage of Ki-67 positive cells was lower in the LV-siRRM2 group (Fig. 7d). The expression levels of RRM2 were lower and the expression levels of STING were higher in the LV-siRRM2 mice (Fig. 7e). These results showed that RRM2 silencing had anti-tumor effects in the nude mouse model.

**Fig. 8:**
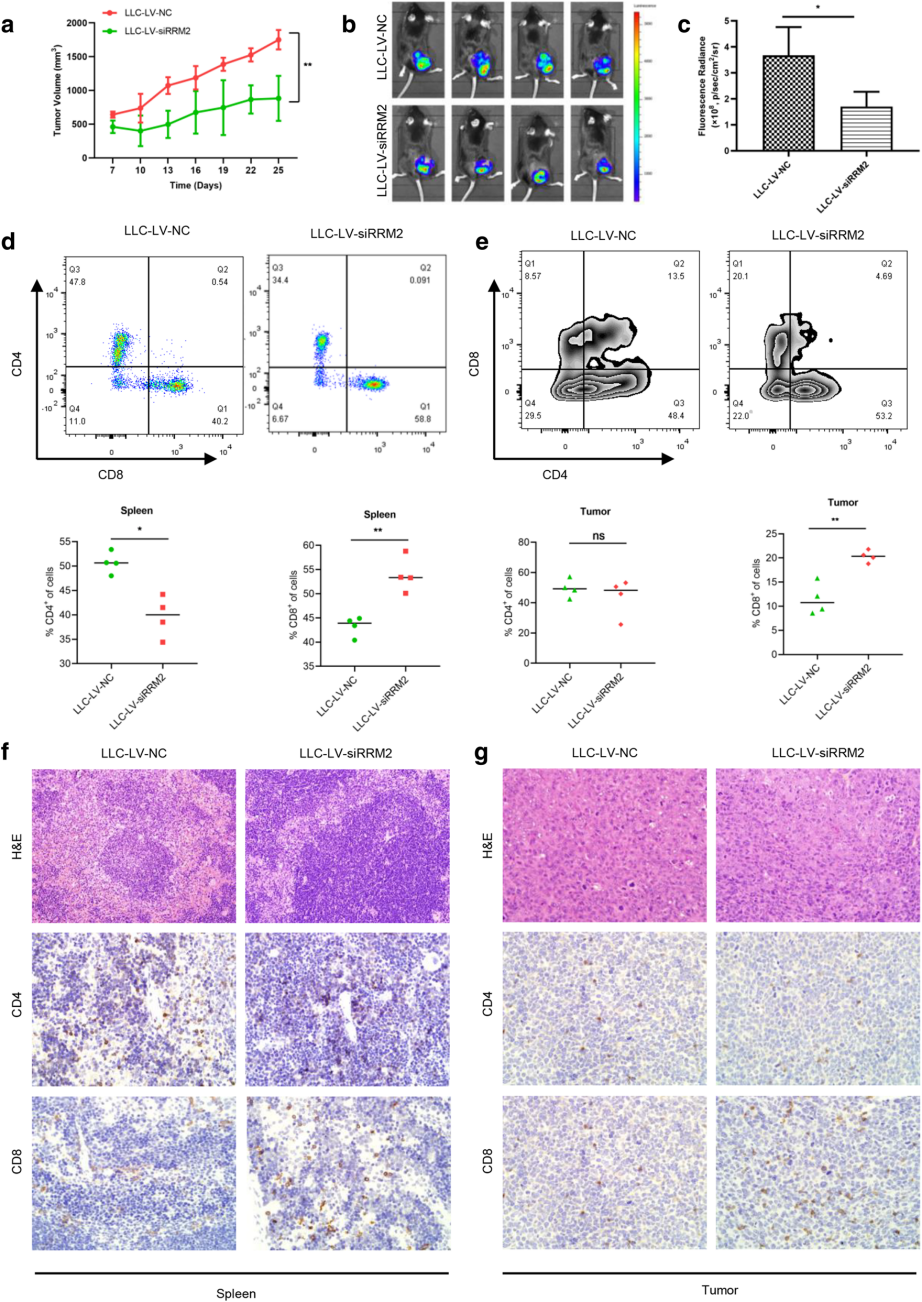
RRM2 silencing has anti-tumor effects and enhances CD8 + T cells infiltrations in Lewis mice model. Lewis mice were injected with LV-NC and LV-siRRM2 infected LLC cells. **a** The tumor volumes were measured every 3 days and depicted in the line chart. **b**, **c** Representative IVIS spectrum imaging for tumor-bearing mice. The fluorescence radiance comparison between LLC-LV-NC and LLC-LV-siRRM2 groups. **d**, **e** Representative flow cytometry of CD4 + and CD8 + T cells in spleens and tumors. Quantitative analysis of CD4 + and CD8 + T cells in spleens and tumors. **f**, **g** Representative image of CD4 and CD8 staining for spleens and tumors by IHC. Scale bar: 100 μm. *p < 0.05, **p < 0.01, ns: not significant

We hypothesized that RRM2 silencing participated in anti-tumor effects by changing tumor microenvironment (TME), the C57BL/6 mice were injected with LV-siRRM2 stable transfected LLC cells. RRM2 deficiency also significantly suppressed tumor growth in C57BL/6 xenograft tumor model (Fig. 8a). The tumor size in the RRM2 silencing group were lower than that in the NC group (Fig. 8b). The fluorescence radiance comparison also showed the same results (Fig. 8c). To further confirm the anti-tumor effects mediated by RRM2 silencing, the flow cytometry was applied to analysis cytotoxic T cells in spleens and tumor tissues. RRM2 deficiency increased CD8 + T cells in both spleen and tumors, and decreased CD4 + T cells in spleen (Fig. 8d, e). The CD4 + and CD8 + T cells in both spleens and tumors were examined with IHC. The results were consistent with flow cytometry analysis (Fig. 8f, g). Taken together, RRM2 silencing drove T cell-related immune responses.

### RRM2 silencing collaborates with radiotherapy to enhance the anti-tumor effects and activation of cGAS/STING pathway

**Fig. 9:**
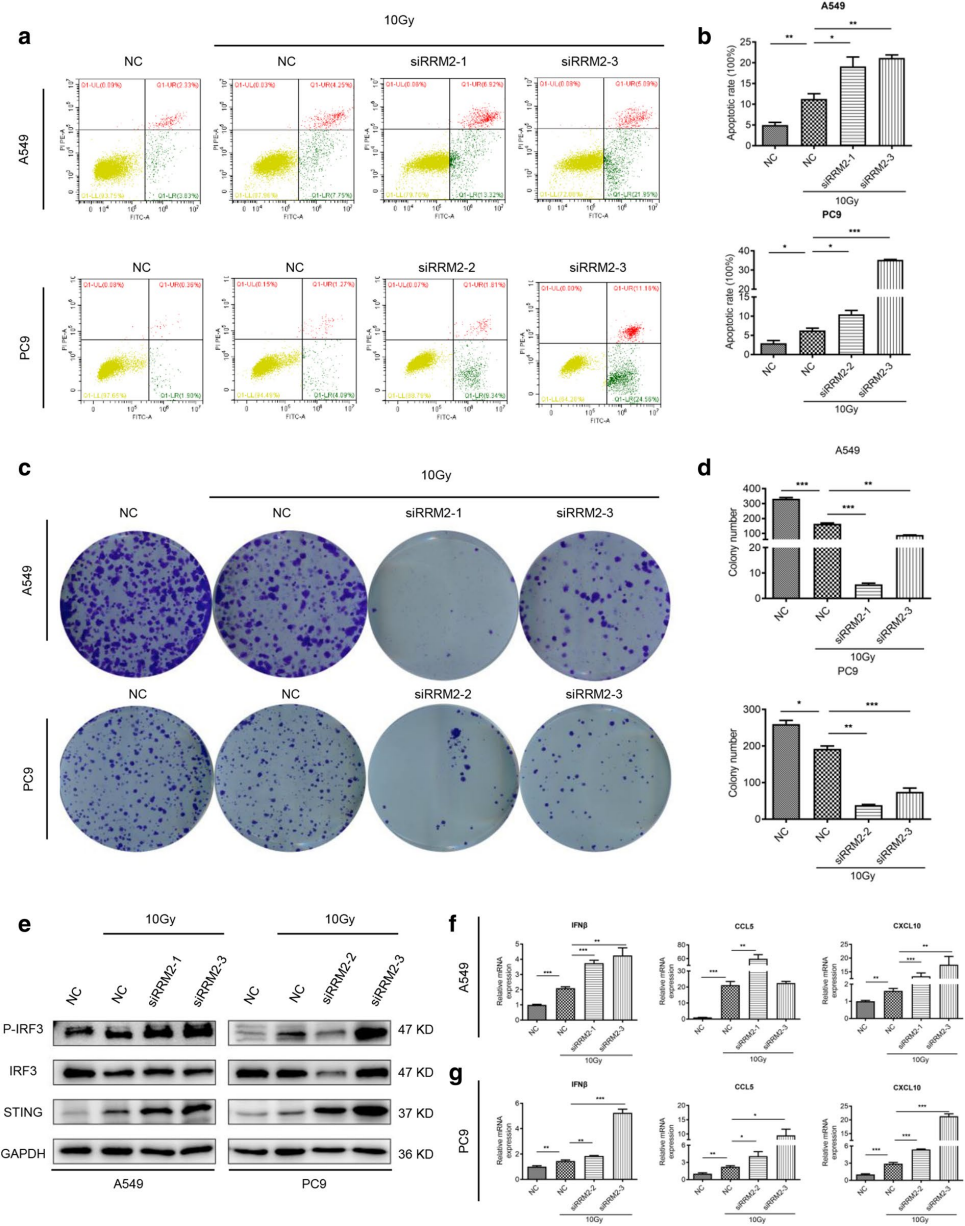
RRM2 silencing collaborates with radiotherapy to enhance anti-tumor effects and activation of cGAS/STING pathway. **a** The synergistic effects of siRRM2 and radiotherapy on apoptosis using flow cytometry in A549 and PC9 cells. **b** The statistical analyses on apoptosis rates. **c** The synergistic effects of siRRM2 and radiotherapy on A549 and PC9 cell colony formation. **d** The statistical analyses on colony formation. **e** Immunoblotting of cGAS/STING pathway-related proteins after siRRM2 treatment and radiotherapy in A549 and PC9 cells. **f** The synergistic effects of siRRM2 and radiotherapy on the mRNA levels of IFNβ, CCL5 and CXCL10 in A549 and PC9 cells. All tests were repeated 3 times. *p < 0.05, **p < 0.01, ***p < 0.001

Our results indicated that RRM2 silencing induced DNA damage and the accumulation of dsDNA in cytoplasm, same as radiotherapy. To investigate whether there is a synergistic effect of RRM2 silencing and radiotherapy, flow cytometry analysis was performed. The results demonstrated that RRM2 silencing collaborated with radiotherapy to enhance LUAD cell apoptosis (Fig. 9a, b). This combined treatment also had a synergistic effect on inhibiting LUAD cell colony formation (Fig. 9c, d). Previous studies proved that radiotherapy activated the cGAS/STING pathway [23–25]. As expected, RRM2 silencing, combined with radiotherapy, further upregulated STING expression and IRF3 phosphorylation in LUAD cells (Fig. 9e). The mRNA levels of IFNβ, CCL5 and CXCL10 were also induced (Fig. 9f, g). These results demonstrated that RRM2 silencing and radiotherapy had synergistic effects on anti-tumor impacts and cGAS/STING pathway activation.

## Discussion

RRM2 (45KD, 389aa) is located at human chromosome 2: (10,122,568–10,211,010) as a rate-limiting enzyme of the synthesis of dNTPs, which were involved in DNA repair and synthesis [9]. Most of RRM2 is located in the cytoplasm and produces dNTPs that diffuse into the nucleus for DNA replication [26]. RRM2 is overexpressed in numerous cancers including nasopharyngeal, ovarian and colorectal cancers [27–29]. Our bioinformatic analysis showed that RRM2 overexpression was correlated with poor prognosis of LUAD patients. Higher RRM2 expression tended to be associated with worse clinicopathological features. Most LUAD patients with higher levels of RRM2 had more advanced TNM and disease stages. Furthermore, RRM2 was an independent prognosis factor for LUAD patients. Given these clinical significances, we further explored the effects of RRM2 both in vivo and in vitro.

Ectopic expression of RRM2 was reported in multiple cancers. In human glioblastoma, RRM2 promoted cell proliferation, migration, and invasion, and reduced apoptosis [14]. In nasopharyngeal carcinoma, RRM2 overexpression enhanced colony formation, as well as cell proliferation, migration and invasion [29]. Suppression of RRM2 inhibited cell growth and invasion in colorectal cancer [30]. Our studies suggested similar results in LUAD cells. RRM2 silencing inhibited cell proliferation and induced S phase arrest simultaneously accompanied by the expression changes of cell cycle associated proteins. Previous studies demonstrated that cyclin E and A were responsible for G1/S transition and S phase progression, respectively [31, 32]. They combined with and activated CDK2 to facilitate S phase entry and progression. Moreover, cyclin E-CDK2 complex also accelerated the degradation of p27, which correlated with the commitment of cells to enter S phase. In our studies, the expression of cyclin E/A-CDK2 complex were significantly upregulated, and p27 was downregulated after RRM2 silencing. Furthermore, cyclin D-CDK4 complex played important roles in G1- to S phase progression [33]. Cyclin D, suppressed only in the S phase [34], was significantly downregulated after RRM2 silencing. CDK4, the catalytic binding partner of the cyclin D, was also remarkably decreased [35]. Eriocitrin was reported to induce S phase arrest and upregulate CDK6 in hepatocellular carcinoma cells [36]. In our study, the expression CDK6 were also upregulated in RRM2-deficient LUAD cells.

Furthermore, our studies indicated that RRM2 silencing inhibited the progress of cell invasion and migration. MMP9 participates in the invasion of tumor cells via promoting the degradation of extracellular matrix proteins including the basement membrane and the surrounding stroma [37]. Moreover, EMT markers (E-cadherin, N-cadherin and vimentin) were reported to be correlated with tumor progression in NSCLC [38, 39]. The switch from E-cadherin to N-cadherin is a strong biomarker of EMT [38]. Our results clearly showed that E-cadherin expression was upregulated. The expression levels of N-cadherin, vimentin and MMP9 were significantly downregulated by RRM2 silencing. These results demonstrated that RRM2 was oncogenic in LUAD.

To further investigate the underlying mechanisms, we conducted bioinformatic analysis and found that the genes in the RRM2 high-expression group were mainly enriched in cell cycle, cytosolic DNA sensing pathway, DNA replication and repair pathways such as p53 signaling pathway. For the RRM2 low-expression group, the enriched signaling pathways were associated with cell adhesion molecules cams and PPAR signaling pathway. In prostate cancer, siRRM2 induced DNA damage accompany with the activation of DNA damage marker γH2AX [12]. Knockdown of RRM2 reduced the HR activity in U2OS cell line [15]. Consistently, we found that RRM2 silencing induced the activation of γH2AX in both A549 and PC9 cells. In addition, RRM2 knockdown led to significant higher accumulation of damaged DNA, clearly indicating a role for RRM2 in response to endogenous DNA damage. According to the bioinformatic results, RRM2 was associated with HR and p53 signaling pathways, and RRM2 knockdown downregulated BRCA1 and upregulated p53.

Abnormal RRM2 degradation induces genome instability such as DSBs. The locations of DSB fragments remain unclear. Our study demonstrated that RRM2 silencing induced DSB fragments accumulation in cytosol. Multiple studies reported that DNA damage sensing by cGAS/STING pathway was critical in pancreatic cancer [40] and NSCLC [41]. Abnormal localization of dsDNA in the cytosol elicits immune responses through the cGAS/STING pathway. STING leads to the phosphorylation of IRF3 that directly contributes to type I IFN transcription and cytokines secretion [7]. Furthermore, the online database TIMER showed that the expression of RRM2 was negatively associated with STING. Consistently, we found that knockdown of RRM2 activated cGAS/STING signaling pathway, and significantly increased the expression of STING, p-STING and p-IRF3. RRM2 silencing had no effects on the cGAS expression levels. Moreover, the downstream molecules of p-IRF3, including IFNβ, CCL5 and CXCL10, were upregulated by RRM2 silencing. STING knockdown partially reversed the activation of cGAS/STING signaling pathway by RRM2 deficiency. In addition, STING knockdown also partly reversed the upregulation of IFNβ, CCL5 and CXCL10 by siRRM2. These results confirmed that RRM2 silencing activated cGAS/STING pathway in a STING partially dependent manner.

Previous studies demonstrated that cGAS/STING pathway initiated anti-tumor immunity via activating and recruiting CD8 + T cells to the TME [42, 43]. To determine whether RRM2 silencing could promote the infiltration of CD8 + T cells in TME, the C57BL/6 mouse model was used, and the results showed that RRM2 silencing increased the infiltration of CD8 + T cells in tumor tissues and spleens.

Increasing evidence demonstrated that radiotherapy activated cGAS/STING signaling pathway based on the radiation-induced DSBs [6, 25]. Previous studies indicated that RRM2 inhibition enhanced radiosensitivity in esophageal cancer [17]. Moreover, knockdown of RRM2 led to apoptosis in A549 cells [44]. Our results also demonstrated that RRM2 silencing collaborated with radiotherapy to promote cell apoptosis and inhibit tumor growth. In addition, they activated cGAS/STING signaling pathway synergistically.

Our studies had several limitations. Our researches were based on animal models and cell experiments. More clinical samples should be collected for translational investigation. In addition, transgenic mice would be an ideal model for further validation of RRM2 function in vivo. More anti-tumor immune effects need to be investigated, such as abscopal effects and immunological memory. Moreover, to confirm the anti-tumor effects of RRM2 silencing were mainly due to cGAS/STING pathway mediated CD8 + T cells infiltrations in vivo, T cells depletion by monoclonal antibody treatment could be a better choice.

## Conclusions

**Fig. 10:**
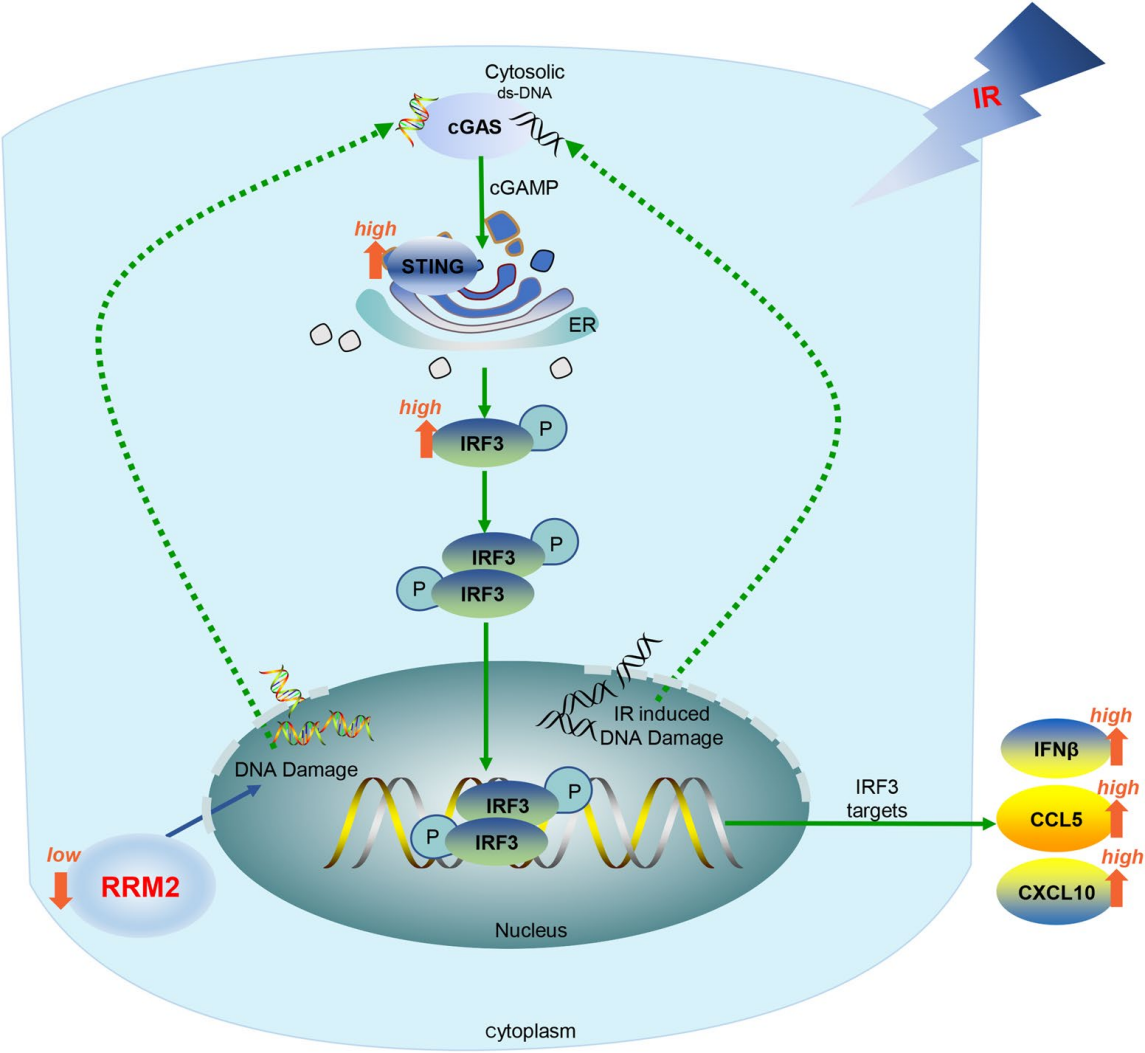
The combination of RRM2 silencing and radiotherapy promotes the activation of cGAS/STING signaling pathway in LUAD cells. Knockdown RRM2 increases DNA damage accompanies by the increase of dsDNA in cytoplasm. Radiation also induces dsDNA accumulation in cytoplasm. The dsDNA fragments activate cGAS and then upregulate STING. Activated STING promotes the phosphorylation of IRF3. This triggers their translocation into the nucleus and results in the increased transcription and secretion of type I IFN genes, including IFNβ, CCL5 and CXCL10

RRM2 deficiency affects various cellular behaviors of LUAD cells, including proliferation inhibition, S phase arrest, migration and invasion inhibition, and DNA damage induction. RRM2 silencing enhances CD8 + T cells infiltrations in TME. Moreover, RRM2 silencing cooperates with radiotherapy to promote cGAS/STING pathway activation (Fig. 10). Therefore, RRM2 may act as a potential target in the diagnosis and therapy of LUAD.

## Supplementary Information

**Additional file 1**: **Fig. S1**. The hoechst staining for mycoplasma testing in A549 and PC9 cells. Scale bar: 50 and 100 μm. **Fig. S2**. The efficiency of siRRM2 was evaluated in LUAD cells. (a) The protein levels of RRM2 in LUAD cell lines (A549, PC9, H1299 and H1975) were detected by immunoblotting. The mRNA levels of RRM2 were measured in A549 (b) and PC9 (c) cells after siRRM2 treatment. The protein levels of RRM2 were examined in A549 (d) and PC9 (e) cells after siRRM2 treatment. (f, g) RRM2 was downregulated in LUAD cells. (h) RRM2 silencing was verified by IF. *p < 0.05, **p < 0.01, ***p < 0.001. **Fig. S3**. The colocalization of dsDNA and cGAS in RRM2-deficient A549 and PC9 cells. Scale bar: 10 μm. **Fig. S4**. RRM2 silencing upregulated the downstream molecules of cGAS/STING signaling pathway. The mRNA levels of IL-6, MX1 and ISG56 were detected by qRT-PCR in RRM2-deficient A549 (a) and PC9 (b) cells. **p < 0.01, ***p < 0.001, ***p < 0.0001. **Fig. S5**. STING deficiency was evaluated in LUAD cells. The mRNA levels of STING were measured in A549 (a) and PC9 (b) cells after siSTING treatment. The protein levels of STING were examined and analyzed in A549 (c) and PC9 (d) cells after siSTING treatment. STING was downregulated by siSTING in LUAD cells. *p < 0.05, **p < 0.01, ***p < 0.001. **Table S1**. Primer sequences used for amplification and the targeting siRNA sequences. **Table S2**. Antibodies used in this research. Table S3. The detailed information about GSEA signaling pathway analysis in both RRM2 high- and low-expression groups.

## Data Availability

The public datasets analyzed in this study can be found in TCGA portal (https://portal.gdc.cancer.gov/). All experimental datasets generated for this study are included in the article/Supplementary Material.

## Abbreviations

LUAD: Lung adenocarcinoma
RRM2: Ribonucleotide reductase regulatory subunit M2
cGAS: Cyclic GMP-AMP synthase
STING: Stimulator of interferon genes
NSCLC: Non-small cell lung cancer
DSBs: Double-strand breaks
dsDNA: Double-stranded DNA
RNR: Ribonucleotide reductase
dNTP: Deoxyribonucleotide triphosphate
HR: Homologous recombination
K-M: Kaplan–Meier
GSEA: Gene Set Enrichment Analysis
siRNA: Small interfering RNA
NC: Negative control
LV: Lentiviruses
qRT-PCR: Quantitative real-time PCR
RT: Room temperature
OD: Optical density
PI: Propidium iodide
IF: Immunofluorescence
H&E: Hematoxylin and eosin
IHC: Immunohistochemistry
ELISA: Enzyme-linked immunosorbent assay
HR: Hazard ratio
EMT: Epithelial mesenchymal transition
IRF3: Interferon regulatory factor 3
p-IRF3: Phospho-IRF3
TME: Tumor microenvironment
