# Correction: RRM2 silencing suppresses malignant phenotype and enhances radiosensitivity via activating cGAS/STING signaling pathway in lung adenocarcinoma

**DOI:** 10.1186/s13578-022-00882-8

**Published:** 2022-09-07

**Authors:** Xueping Jiang, Yangyi Li, Nannan Zhang, Yanping Gao, Linzhi Han, Shuying Li, Jiali Li, Xingyu Liu, Yan Gong, Conghua Xie

**Affiliations:** 1 Department of Radiation and Medical Oncology, Zhongnan Hospital of Wuhan University, Wuhan, 430071 Hubei China; 2 Department of Biological Repositories, Zhongnan Hospital of Wuhan University, Wuhan, 430071 Hubei China; 3 Tumor Precision Diagnosis and Treatment Technology and Translational Medicine, Hubei Engineering Research Center, Zhongnan Hospital of Wuhan University, Wuhan, 430071 Hubei China; 4 Hubei Key Laboratory of Tumor Biological Behaviors, Zhongnan Hospital of Wuhan University, Wuhan, 430071 Hubei China; 5 Hubei Cancer Clinical Study Center, Zhongnan Hospital of Wuhan University, Wuhan, 430071 Hubei China

## Correction: Cell Biosci (2021) 11:74 https://doi.org/10.1186/s13578-021-00586-5

In this article [1], the representative images of CD4 and CD8 staining for tumor IHC were carelessly repeated in Fig. 8. The corrected Fig. 8 should have appeared as shown in this correction.

**Fig. 8:**
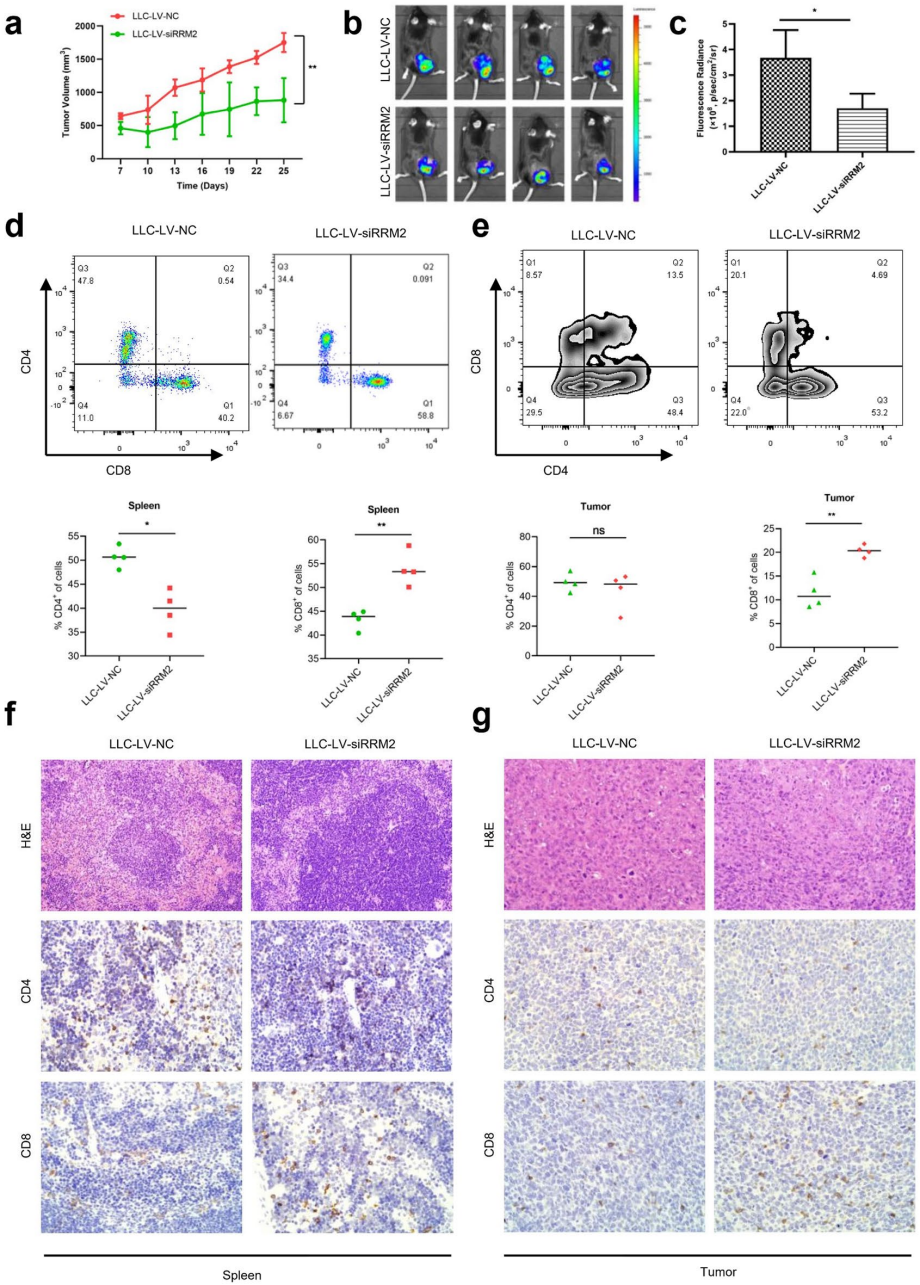
RRM2 silencing has anti-tumor effects and enhances CD8 + T cells infiltrations in Lewis mice model. Lewis mice were injected with LV-NC and LV-siRRM2 infected LLC cells. **a** The tumor volumes were measured every 3 days and depicted in the line chart. **b**, **c** Representative IVIS spectrum imaging for tumor-bearing mice. The fluorescence radiance comparison between LLC-LV-NC and LLC-LV-siRRM2 groups. **d**, **e** Representative fow cytometry of CD4 + and CD8 + T cells in spleens and tumors. Quantitative analysis of CD4 + and CD8 + T cells in spleens and tumors. **f**, **g** Representative image of CD4 and CD8 staining for spleens and tumors by IHC. Scale bar: 100 μm. *p < 0.05, **p < 0.01, ns: not significant
