# Supplementary material for: RRM2 silencing suppresses malignant phenotype and enhances radiosensitivity via activating cGAS/STING signaling pathway in lung adenocarcinoma
Source: Cell Biosci. 2021 Apr 15;11:74. doi: 10.1186/s13578-021-00586-5 (PMC8051110; doi:10.1186/s13578-021-00586-5)
Supplement: Supplementary file 1 — Additional file 1: Fig. S1. The hoechst staining for mycoplasma testing in A549 and PC9 cells. Scale bar: 50 and 100 μm. Fig. S2. The efficiency of siRRM2 was evaluated in LUAD cells. (a) The protein levels of RRM2 in LUAD cell lines (A549, PC9, H1299 and H1975) were detected by immunoblotting. The mRNA levels of RRM2 were measured in A549 (b) and PC9 (c) cells after siRRM2 treatment. The protein levels of RRM2 were examined in A549 (d) and PC9 (e) cells after siRRM2 treatment. (f, g) RRM2 was downregulated in LUAD cells. (h) RRM2 silencing was verified by IF. *p < 0.05, **p < 0.01, ***p < 0.001. Fig. S3. The colocalization of dsDNA and cGAS in RRM2-deficient A549 and PC9 cells. Scale bar: 10 μm. Fig. S4. RRM2 silencing upregulated the downstream molecules of cGAS/STING signaling pathway. The mRNA levels of IL-6, MX1 and ISG56 were detected by qRT-PCR in RRM2-deficient A549 (a) and PC9 (b) cells. **p < 0.01, ***p < 0.001, ***p < 0.0001. Fig. S5. STING deficiency was evaluated in LUAD cells. The mRNA levels of STING were measured in A549 (a) and PC9 (b) cells after siSTING treatment. The protein levels of STING were examined and analyzed in A549 (c) and PC9 (d) cells after siSTING treatment. STING was downregulated by siSTING in LUAD cells. *p < 0.05, **p < 0.01, ***p < 0.001. Table S1. Primer sequences used for amplification and the targeting siRNA sequences. Table S2. Antibodies used in this research. Table S3. The detailed information about GSEA signaling pathway analysis in both RRM2 high- and low-expression groups. [file 13578_2021_586_MOESM1_ESM.pdf]

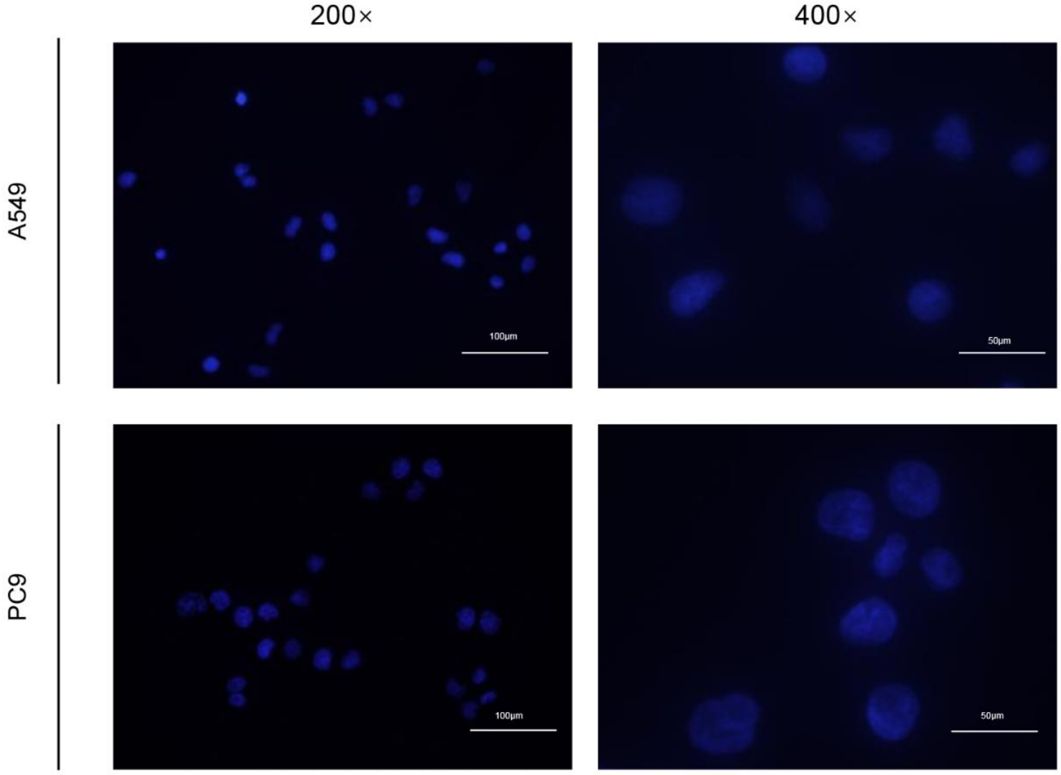

Fig. S1. The hoechst staining for mycoplasma testing in A549 and PC9 cells. Scale bar: 50 and 100 μm.

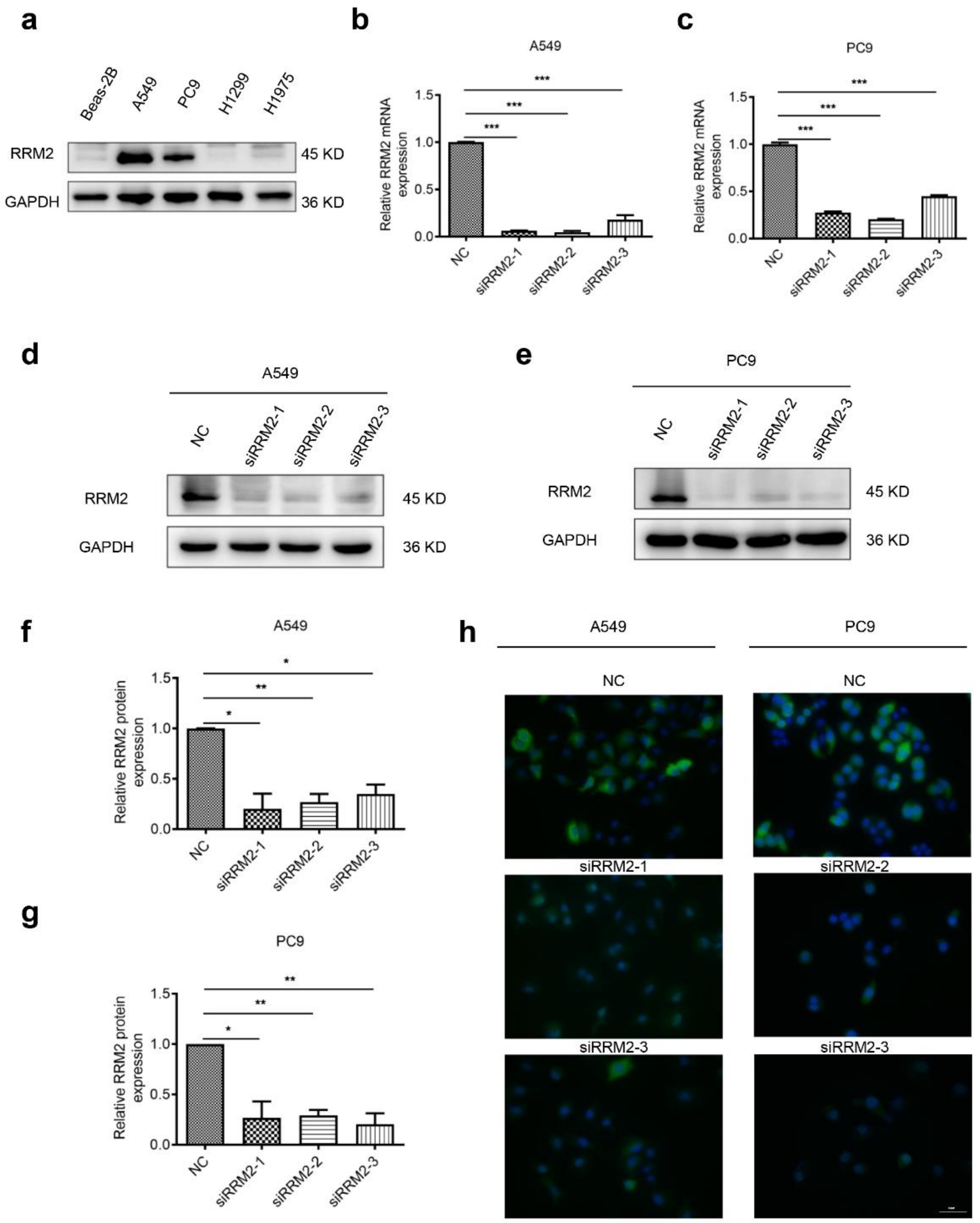

Fig. S2. The efficiency of siRRM2 was evaluated in LUAD cells. (a) The protein levels of RRM2 in LUAD cell lines (A549, PC9, H1299 and H1975) were detected by immunoblotting. The mRNA levels of RRM2 were measured in A549 (b) and PC9 (c) cells after siRRM2 treatment. The protein levels of RRM2 were examined in A549 (d) and PC9 (e) cells after siRRM2 treatment. (f, g) RRM2 was downregulated in LUAD cells. (h) RRM2 silencing was verified by IF. *$p < 0.05$, **$p < 0.01$, ***$p < 0.001$.

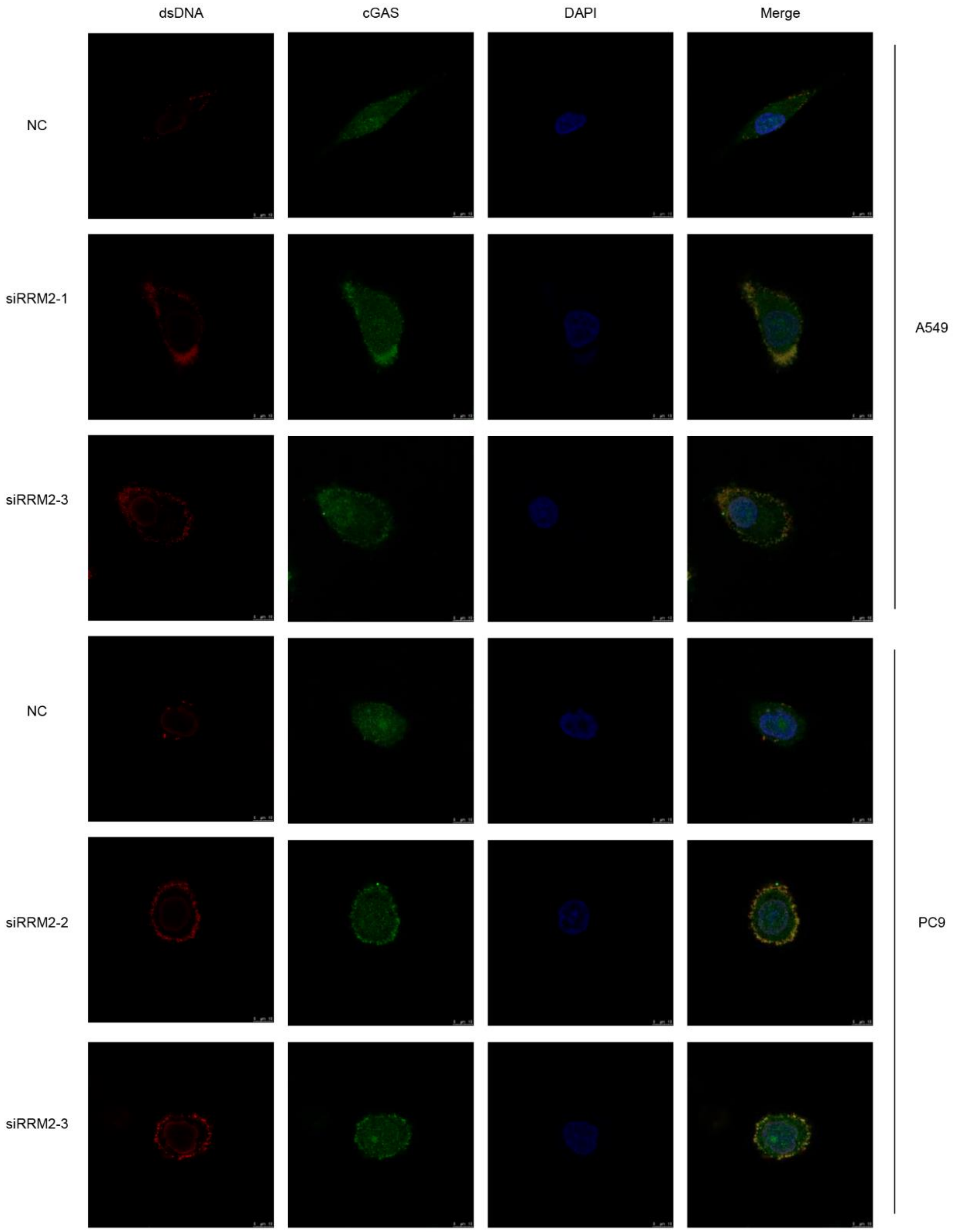

Fig. S3. The colocalization of dsDNA and cGAS in RRM2-deficient A549 and PC9 cells. Scale bar: 10 μm.

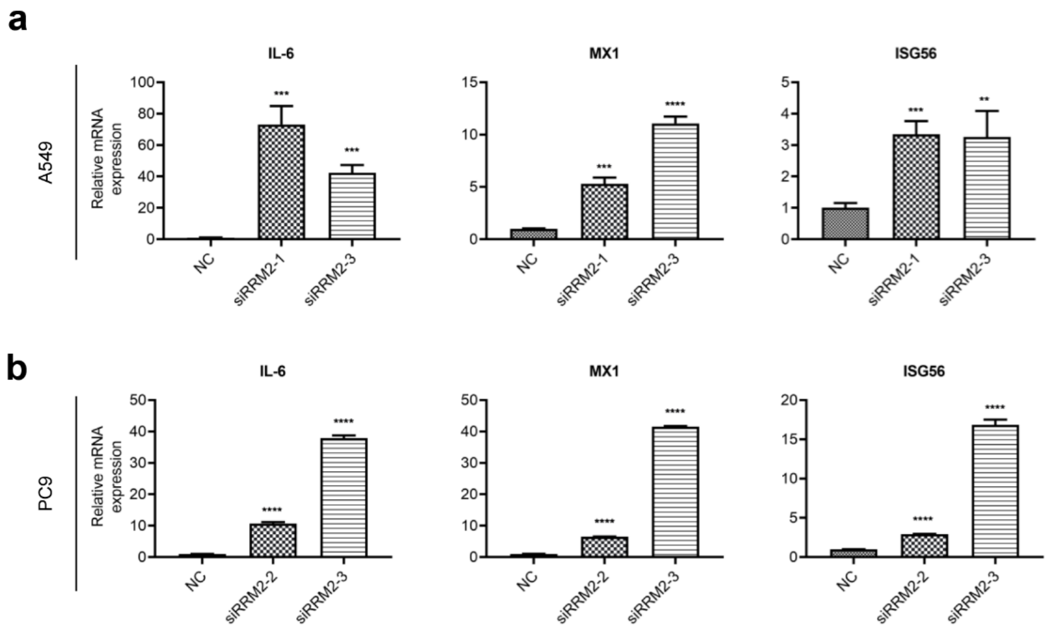

Fig. S4. RRM2 silencing upregulated the downstream molecules of cGAS/STING signaling pathway. The mRNA levels of IL-6, MX1 and ISG56 were detected by qRT-PCR in RRM2-deficient A549 (a) and PC9 (b) cells. **$p < 0.01$, ***$p < 0.001$, ***$p < 0.0001$.

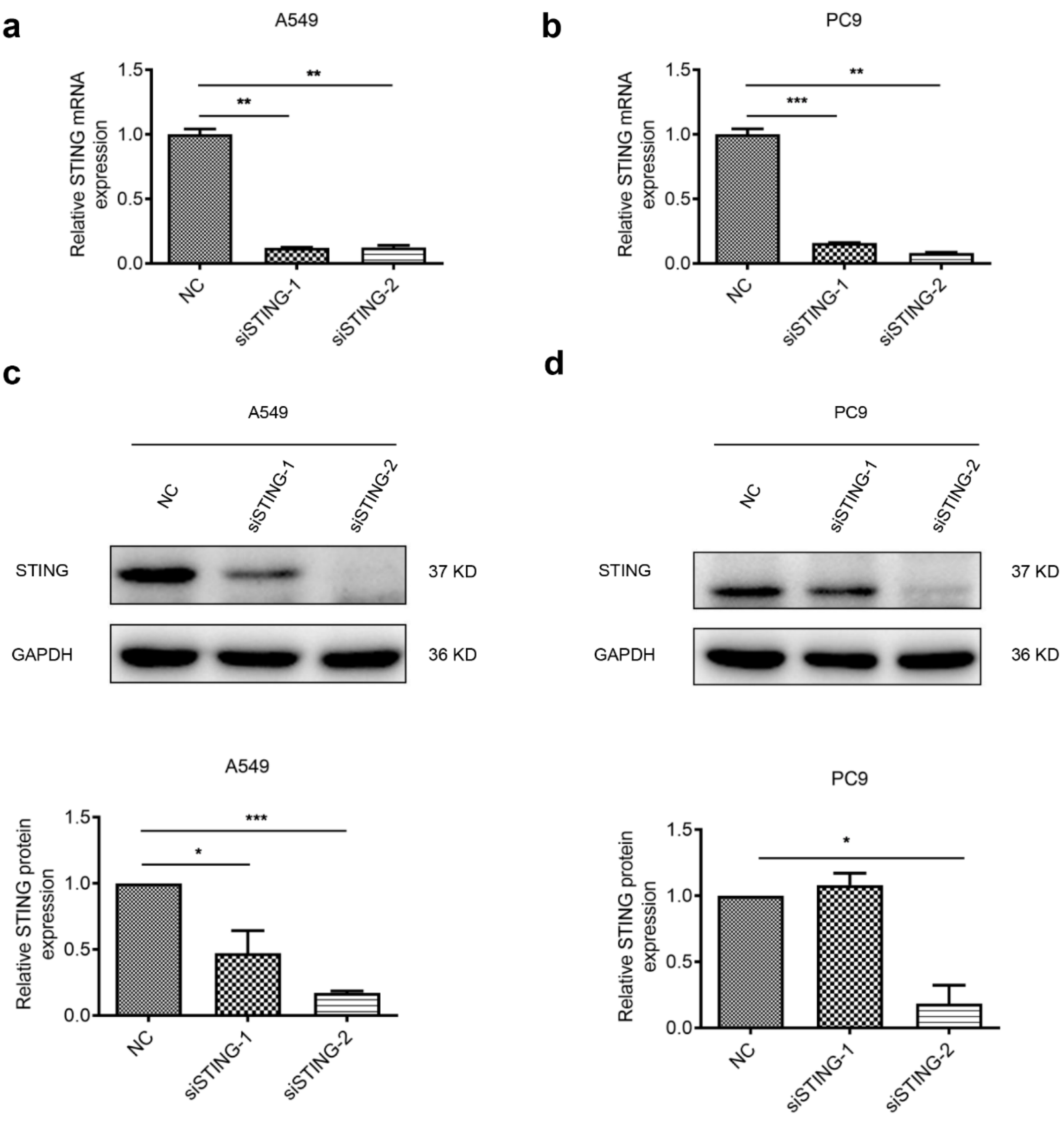

Fig. S5. STING deficiency was evaluated in LUAD cells. The mRNA levels of STING were measured in A549 (a) and PC9 (b) cells after siSTING treatment. The protein levels of STING were examined and analyzed in A549 (c) and PC9 (d) cells after siSTING treatment. STING was downregulated by siSTING in LUAD cells. *$p < 0.05$, **$p < 0.01$, ***$p < 0.001$.

Table S1. Primer sequences used for amplification and the targeting siRNA sequences.

**Gene**	**Sequences or target sequence (5'→3')**
GAPDH Fp	GACAAGCTTCCCGTTCTCAG
GAPDH Rp	GAGTCAACGGATTTGGTGGT
RRM2 Fp	GTGGAGCGATTTAGCCAAGAA
RRM2 Rp	CACAAGGCATCGTTTCAATGG
STING Fp	GCTGCTGTCCATCTATTTCTACT
STING Rp	GCCGCAGATATCCGATGTAATA
IFNβ Fp	TTGTTGAGAACCTCCTGGCT
IFNβ Fp	TGACTATGGTCCAGGCACAG
CCL5 Fp	CGCTGTCATCCTCATTGCTA
CCL5 Rp	CCAGACTTGCTGTCCCTCTC
CXCL10 Fp	CTGTACGCTGTACCTGCATCA
CXCL10 Rp	TTCTTGATGGCCTTCGATTC
IL-6 Fp	GCCGCATCGCCGTCTCCTAC
IL-6 Rp	CCTCAGCCCCCTCTGGGGTC
MX1 Fp	GTCCCGGATCTGACTCTAAT
MX1 Rp	GTCTCCTGCCTCTGGATGTA
ISG56 Fp	TCTCAGAGGAGCCTGGCTAA
ISG56 Rp	TGACATCTCAATTGCTCCAG
siRRM2-1	CCAUCGAGUACCAUGAUAUTT
siRRM2-2	GGAGCGAUUUAGCCAAGAATT
siRRM2-3	GCACUCUAAUGAAGCAAUATT
siSTING-1	CAGCGGCUGUAUAUUCUCCUCCCTT
siSTING-2	GGUCAUAUUACAUCGGAUATT

Table S2. Antibodies used in this research.

Antibody	Company	Catalog number
CyclinA1	Abcam	ab53699
CyclinD1	Proteintech	26939-1-AP
CyclinE1	Proteintech	11554-1-AP
CDK2	Proteintech	10122-1-AP
CDK4	Proteintech	11026-1-AP
CDK6	Proteintech	14052-1-AP
P27	Proteintech	25614-1-AP
Ki-67	Proteintech	27309-1-AP
E-Cadherin	Proteintech	20874-1-AP
N-Cadherin	Proteintech	22018-1-AP
Vimentin	Proteintech	10366-1-AP
MMP9	Proteintech	10375-2-AP
γH2AX	ABclonal	AP0099
BRCA1	Proteintech	22362-1-AP
P53	Proteintech	10442-1-AP
dsDNA	Abcam	ab27156
p-IRF3	Cell Signaling Technology	37829
IRF3	Proteintech	11312-1-AP
STING	Proteintech	19851-1-AP
RRM2	ABclonal	A5255
GAPDH	Proteintech	10494-1-AP
Ms CD45 PerCP-Cy5.5	BD Pharmingen	550994
Ms CD3 APC-Cy7	BD Pharmingen	560590
PE-Cy™7 Rat Anti-Mouse CD4	BD Pharmingen	552775
Fluor® 647 Rat Anti-Mouse CD8a	BD Pharmingen	557682
Fluor®488 Donkey Anti-Rabbit IgG	Antgene	ANT024
Dylight 549 Goat Anti-Mouse IgG	Abbkin	A23310
HRP-conjugated Goat Anti-Rabbit IgG	Proteintech	SA00001-2
HRP-conjugated Goat Anti-Mouse IgG	Proteintech	SA00001-1

Table S3. The detailed information about GSEA signaling pathway analysis in both RRM2 high- and low-expression groups.

	NAME	ES	NES	NOM p-val	FDR q-val
High-expression	KEGG_CELL_CYCLE	0.8357297	2.9514272	0	0
	KEGG_CYTOSOLIC_DNA_SENSING_PATHWAY	0.49036065	1.5245888	0.01055807	0.03950674
	KEGG_DNA_REPLICATION	0.9397333	2.6876562	0	0
	KEGG_HOMOLOGOUS_RECOMBINATION	0.8814975	2.3941972	0	0
	KEGG_MISMATCH_REPAIR	0.9123358	2.427915	0	0
	KEGG_P53_SIGNALING_PATHWAY	0.6609586	2.1234787	0	0
	KEGG_PATHWAYS_IN_CANCER	0.43633893	1.6982678	0	0.008151803
	KEGG_TOLL_LIKE_RECEPTOR_SIGNALING_PATHWAY	0.46138301	1.5902151	0.002770083	0.024162317
Low-expression	KEGG_CELL_ADHESION_MOLECULES_CAMS	-0.4048367	-1.5971215	0.004149378	0.040985964
	KEGG_COMPLEMENT_AND_COAGULATION_CASCADES	-0.4555781	-1.6627984	0	0.029792212
	KEGG_INTESTINAL_IMMUNE_NETWORK_FOR_IGA_PRODUCTION	-0.547823	-1.8184946	0	0.008313705
	KEGG_PPAR_SIGNALING_PATHWAY	-0.4447429	-1.6007236	0.0032258064	0.042671133
